# Origin, evolution, and divergence of plant class C GH9 endoglucanases

**DOI:** 10.1186/s12862-018-1185-2

**Published:** 2018-05-30

**Authors:** Siddhartha Kundu, Rita Sharma

**Affiliations:** 1 Department of Biochemistry, Government of NCT of Delhi, Dr. Baba Saheb Ambedkar Medical College & Hospital, New Delhi, 110085 India; 2 Crop Genetics and Informatics Group, School of Computational and Integrative Sciences, Jawaharlal Nehru University, New Delhi, 110067 India

**Keywords:** Cellulase, Cellulose, Glycoside hydrolase, GH9, Endoglucanases, Phylogenetics

## Abstract

**Background:**

Glycoside hydrolases of the GH9 family encode cellulases that predominantly function as endoglucanases and have wide applications in the food, paper, pharmaceutical, and biofuel industries. The partitioning of plant GH9 endoglucanases, into classes A, B, and C, is based on the differential presence of transmembrane, signal peptide, and the carbohydrate binding module (CBM49). There is considerable debate on the distribution and the functions of these enzymes which may vary in different organisms. In light of these findings we examined the origin, emergence, and subsequent divergence of plant GH9 endoglucanases, with an emphasis on elucidating the role of CBM49 in the digestion of crystalline cellulose by class C members.

**Results:**

Since, the digestion of crystalline cellulose mandates the presence of a well-defined set of aromatic and polar amino acids and/or an attributable domain that can mediate this conversion, we hypothesize a vertical mode of transfer of genes that could favour the emergence of class C like GH9 endoglucanase activity in land plants from potentially ancestral non plant taxa. We demonstrated the concomitant occurrence of a GH9 domain with CBM49 and other homologous carbohydrate binding modules, in putative endoglucanase sequences from several non-plant taxa. In the absence of comparable full length CBMs, we have characterized several low strength patterns that could approximate the CBM49, thereby, extending support for digestion of crystalline cellulose to other segments of the protein. We also provide data suggestive of the ancestral role of putative class C GH9 endoglucanases in land plants, which includes detailed phylogenetics and the presence and subsequent loss of CBM49, transmembrane, and signal peptide regions in certain populations of early land plants. These findings suggest that classes A and B of modern vascular land plants may have emerged by diverging directly from CBM49 encompassing putative class C enzymes.

**Conclusion:**

Our detailed phylogenetic and bioinformatics analysis of putative GH9 endoglucanase sequences across major taxa suggests that plant class C enzymes, despite their recent discovery, could function as the last common ancestor of classes A and B. Additionally, research into their ability to digest or inter-convert crystalline and amorphous forms of cellulose could make them lucrative candidates for engineering biofuel feedstock.

**Electronic supplementary material:**

The online version of this article (10.1186/s12862-018-1185-2) contains supplementary material, which is available to authorized users.

## Background

Glycoside hydrolase 9 (GH9) endoglucanases utilize water (EC3.x.y.z) to cleave the glycoside (1 → 4) or (1 → 3) bonds between repeated monomeric *β*(*D*)-glucopyranose units of cellulose and comprise sequences from all major kingdoms of life [1, 2]. GH9 endoglucanases in land plants were previously clustered into classes A and B on the basis of the presence/ absence of transmembrane (TM) and/ or signal peptide (SP) sub regions [1, 2]. The abundantly present amorphous cellulose is enzymatically amenable to digestion, and is the de facto substrate for these enzymes. However, an editing/ modifying function for crystalline cellulose has been ascribed to class A endoglucanases, either exclusively or in association with the cellulosome [3–5]. The discovery and further characterization of a carbohydrate binding module (CBM49) at the C-termini of previously annotated GH9 endoglucanases (classes A and B) in *Solanum lycopersicum, Oryza sativa, Arabidopsis thaliana,* and *Nicotiana tabacum* conferred, on this family, catalytic competency for crystalline cellulose [6–8]. The hydrogen-bond stabilized crystalline cellulose, is the preferred substrate for bacteria, fungi, archaea, and protists, organisms which predate the emergence of green land plants by several millions of years [9–14]. The discovery, therefore, that a subset of plant GH9 endoglucanases could utilize crystalline cellulose as its cognate substrate raises fundamental questions not only on the evolution and ancestry of plant GH9 endoglucanases, but also the functional relevance of an additional hydrolase with a hitherto novel spectrum of catalytic activity.

**Fig. 1:**
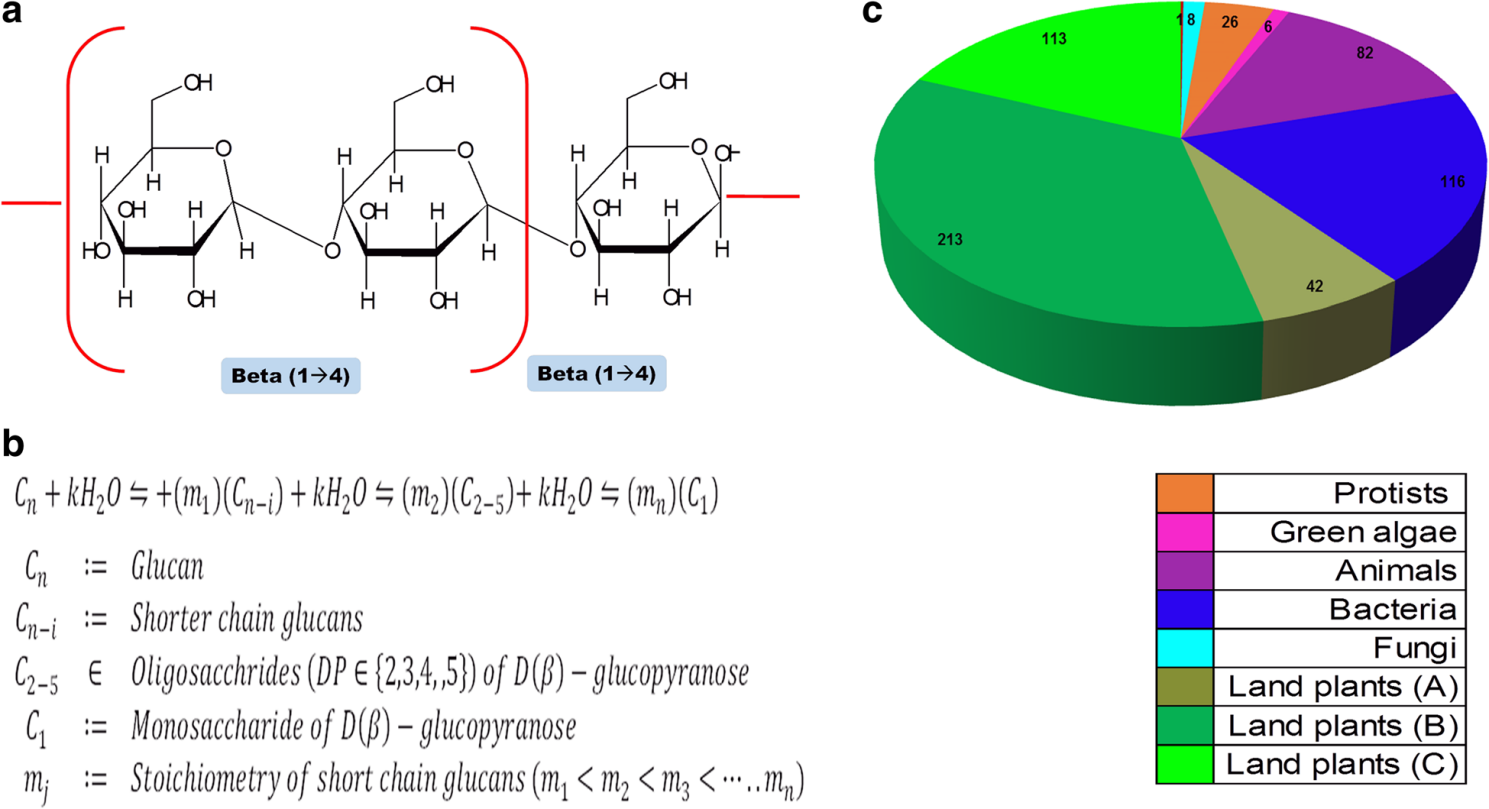
Taxonomic distribution and analysis of the GH9 domain in putative endoglucanse sequences. **a** Molecular structure of cellulose with repeating units of D-glucopyranose linked by a *β*(1 → 4) glycosidic bond. The liberated mono- or oligosaccharides either retain the *β*-hydroxyl group (retaining), or are inverted (*α*-hydroxyl) after transformation, **b** Generic reaction mechanism of hydrolytic GH9 endoglucanase (*EC* 3.2.1.4) mediated transformation of cellulose into simpler oligo- and/or mono-saccharides, **c** Alignment compatible sequences of GH9 domains from putative GH9 endoglucanases across all taxa (*n*_1_ = 607). Abbreviations: GH9, glycoside hydrolase; EC, enzyme commission

Cellulose, is a straight chain polymer of repeating units of *β*(1 → 4) linked D-glucopyranose residues and consists of microcrystalline (*I*_*α*_, *I*_*β*_) and amorphous (*I*_*α*_*am*, *I*_*β*_*am*) regions (Fig. 1a and b). This heterogeneous distribution is dictated by the presence of a rich inter-and intra-fibrillar hydrogen bond network. Whilst, the paucity of hydrogen bonds in the former facilitates enzymatic cleavage, the ordered structure of the latter, imposes constraints on the activity profile of plant GH9 endoglucanases. Natural cellulose is rarely pure (*Gossypium* spp., 90%), and is frequently found in association with other carbohydrates (hemicellulose) and/ or other macromolecules (lipids, proteins). The presence of these complexes would also imply, reciprocally, the existence of mixed function endo- and exo-glucanases acting in tandem with biosynthetic catalysts to modulate the composition of the encompassing cell wall matrix/ capsule/ coat [15–17]. Observations by several investigators suggest a correlation between exhibited function with the occurrence of sequence homology or manifested enzymatic activity. Thus, despite the proximity of divergence between multicellular green algae and primitive land plants 470 − 480 *Million years ago* (*Mya*), homologous GH9 endoglucanase sequences are either completely absent or at best partial and fragmented in unicellular members (*Chlamydomonas reinhardtii, Volvox carteri*) [16, 17]. In contrast, bacteria (≅3200 − 3950 *Mya*), archaea (≅390 − 1350 *Mya*), protists (≅2000 − 3000 *Mya*), fungi (≅1000 − 1500 *Mya*), and some animals (180 − 670 *Mya*) not just possess sequences with ascribable GH9 endoglucanase activity of crystalline cellulose, but also a demonstrable and relevant function (Table 1) [18–40]. These include modulation of sporulation (*Dictyostelium spp.*, clostridiales, bacillales), host-pathogen interactions (fungi, nematodes, protists, plants), repair and survival (Euryarchaea), and preventive desiccation (bacteria, *Dictyostelium spp*.) [15, 41–49]. Genomic evidence of GH9 endoglucanases in some animals (marine invertebrates, termites, arthropods, parasitic and saprophytic nematodes), in the absence of demonstrable function, was postulated to have occurred during phases of co-infection with gastrointestinal and oral microbiota [15, 42, 44, 45, 50–54]. However, the confirmed presence in numerous other animals, similarity in substrate and reaction chemistry, and sequence conservation, along with supporting laboratory data has refuted much of this horizontal transfer mode of gene transfer [15, 41, 42, 44, 45, 55–57]. Davison and Blaxter suggested a single origin of GH9 genes based on monophyly in the phylogenetic tree and conserved intron positions [55].

**Table 1:** Literature based divergence rates of taxa utilized for calibrating the time trees

Taxa	Divergence (*Mya*)
Bacteria	3200–3950
Protists	1600–3000
Archaea	500–2500
Animals	1000
Crustacea	511
Insects	396
*C.intestinalis*	180
Chordates	542
Arthropoda	540
Fungi	1500
Green algae	500–2000
Bryophytes	470–475
Tracheophytes	395–425
Land Plants	
Monocots	90–141
Eudicots	90–141
Rosids	108–117
Asterids	107–117

*Abbreviations*: *Mya* Millions of years

In land plants (*Viridiplantae*), the activity profile of GH9 endoglucanases on cellulose, correlates, in part, with their distribution, as well as the purported roles in growth, development, flowering, and seed germination [16]. The carbohydrate binding modules/ domains (*n* = 64), are sequences 40 − 200 *aa* in length, and despite being intrinsically non catalytic can facilitate the hydrolytic cleavage of the glycosidic linkage [47]. Unlike the C-terminally localized CBM49 of plant GH9 endoglucanases, different CBMs favouring the activity on crystalline cellulose in bacteria, fungi, protists, animals, and possibly archaea and green algae are distributed throughout the length of the sequence [16]. The presence of one or more TM regions also suggests that at least in plants cellulose metabolism may occur in clusters of (biosynthetic, degrading enzymes) and be localized at the membrane itself [4, 5]. The presence of signal peptide regions, in contrast, posits that these enzymes may be secreted and digest cellulose extracellularly. Such a mechanism might benefit fungal pathogens of plants, may be deployed by termites, and participate in glucose extraction in ruminants as well [15, 42, 44, 48]. The proportion of sequences that exhibit class B and C activity is subject to much debate. Whilst, a simple sequence similarity suggests a preponderance of class B members, complex classification schema using hidden markov models (HMM) and artificial neural networks (ANN) indicates a marginally greater number of putative class C GH9 endoglucanases in primary transcript data from sequenced land plants [16, 58–60].

The potential importance of class C enzymes in biomass conversion notwithstanding, a paradigm shift in the chemical nature of cellulose, the inconsistencies in the numbers observed between predicted and observed members, and a conserved reaction chemistry in extant non plant taxa, suggest that plant class C GH9 endoglucanases may predate classes A and B enzymes [16, 58–61]. Here, we attempt to resolve some of these queries by investigating the origins, evolution, and subsequent divergence of the GH9 domain in putative plant endoglucanase sequences, with particular emphasis on the contribution of class C members. The role of the aromatic (W/ Y / F) and polar uncharged (S/T/N/Q) is critical to the functioning of endoglucanases in the presence and absence of well-defined CBMs, and, in the presence of low complexity regions their incorporation into the GH9 domain might constitute the only measure of approximating the CBM49 [62–64]. These residues despite being non-catalytic themselves have been shown to confer the capacity on the encompassing enzymes to discriminate between related ligands (cellulose/ X, X = {xylose, lignin, chitin; β-1,3/β-1,4), effect and in some cases even the binding affinity for a cognate substrate, contribute to processivity and thermal stability, and interestingly introduce catalytic competency [62–78]. We utilize a combination of phylogenetic analysis, pattern approximation, identification, distribution analysis, and residue mapping of the CBM49 to investigate the emergence of crystalline cellulose digesting activity in land plants. Finally, we complement these analyses by examining the presence and distribution of transmembrane and signal peptide regions in vascular land plants, and the possible routes by which endoglucanase sequences with putative class C activity could contribute to the emergence of sequences with novel functionality.

## Methods

### Collation, annotation, and domain extraction of GH9 endoglucanases

Sequences of putative GH9 endoglucanases were downloaded from the publically available databases National Center for Biotechnology Information (NCBI; http://www.ncbi.nlm.nih.gov) and Carbohydrate-active enzymes (CAZy; http://www.cazy.org/) [16, 79, 80]. Sequences of green land plants (*Viridiplantae*) utilized for this analysis were downloaded from Phytozome (https://phytozome.jgi.doe.gov/pz/portal.html), extensively curated, and classified into classes A, B, and C as described previously [16]. Annotation for non-plant GH9 endoglucanases was in accordance with the schema adopted by dbCAN (Carbohydrate enzyme annotation; http://csbl.bmb.uga.edu/dbCAN) [81]. The pooled sequences were filtered on the basis of their contribution to a compatible multiple sequence alignment (MSA) and the presence of a single GH9 domain as determined by MEGA7.0 (Molecular evolutionary genetic analysis, local installation) and the SMART (Simple modular architecture research tool) server [82–84]. Exclusion criteria for this preliminary data were: a) an indeterminable MSA, b) the complete absence of a demonstrable GH9 domain, c) more than one GH9 domain ((*GH*9)_*x*_ : *x* > 1) in the same sequence, and d) presence of a concomitant GH domain other than GH9 ((*GH*9 ∧ *GHx*) : *x*∈[1, 8] ∧ [10 − 130]). Amino acids at the start and end positions of the GH9 domains were noted and extracted (*n*_1_) using in-house developed PERL scripts (Additional file 1: Text S1, Additional file 2: Text S3, and Additional file 3: Text S4). Here, the final set of compatible sequences of the GH9 domains (*n*_1*A*_), pattern selected GH9 domains (*n*_1*B*_, *n*_1*C*_), pattern selected and GH9 encompassing full length sequences (*n*_2_), CBM49/ CBM49-like sequences of land plants (*n*_3*X*_ = *n*_*LPSX*_; *X* = {*A*, *B*, *C*}) comprised the datasets utilized in this study. The distinct and delineable CBM49 from putative class C GH9 endoglucanases was similarly isolated and comprised (*n*_3*C*_ = *n*_*LPSC*_) (Additional file 4: Text S2). The amino acid content of the extricated GH9 and CBM49 domains were assessed using PIR (Protein information server, http://pir.georgetown.edu) and categorized on the basis of side chain content into those with hydrophobic side chains (HSC), aromatic amino acids (AAA), polar uncharged (PUC), polar charged acidic (PCA), and polar charged basic (PCB). The GH9 domains were used for phylogenetic analysis and time tree estimation (*n*_1*A*_), CBM49 was utilized for pattern analysis and motif approximation (*n*_3*C*_), and CBM49-like full length sequences from plant and non-plant taxa were utilized for assessing relevant bioinformatics indices (*n*_1*B*_, *n*_1*C*_) (Additional file 1: Text S1, Additional file 4: Text S2, Additional file 2: Text S3 and Additional file 3: Text S4).

### Model selection, phylogenetic analysis, and time tree estimation

Multiple sequence alignments (MSA) of the extracted GH9 domains and the CBM49/ CBM49-like in land plants were generated using the default parameters (gap opening = gap extension = 10), with gap opening penalties of 0.1 (pairwise alignment) and 0.2 (MSA), a divergence cut off of 20%, and the BLOSUM62 set of matrices (Additional file 5: Table S1, Additional file 1: Text S1 and Additional file 3: Text S4) [85, 86]. This was chosen to account for the purported domain distribution of classes A, B, and C among the various taxa. Sequences were deemed compatible if and only if their pairwise alignments were free from errors as determined by the distance matrix computed by MEGA7.0. The top scoring amino acid substitution models for the aforementioned MSAs was selected amongst all (*n* = 56) using the Akaike information criteria corrected (min(*AICc*)) and the Bayesian information criteria (min(*BIC*)) as indices (Additional file 6: Table S3). BEAST v2.4.7 (Bayesian evolutionary analysis by sampling trees) and the accompanying software suite (FigTree v1.4.3, DensiTree, Tracer v1.6, TreeAnnotator) was utilized to infer the date and visualize a maximum clade credibility tree with median heights, and tabulate descriptive statistics after the posterior probabilities converged (Tables 2 and 3; Additional file 7: Table S4) [87–89]. Whilst, the age of the node and the branch times of the clades were inferred directly (*Mya*), support was denoted as the posterior probabilities (*PP*%) and bootstrap values (*n* = 1000) by maximum likelihood (*ML*%), i.e., *support* = *PP* % , *ML*%, (FigTree v1.4.3). Whilst, the selection of the root for evaluating the evolution of the GH9 domain (parent of the bacterial clade), was based on fossil records that suggested that bacteria were amongst the earliest forms of life (≈3170 − 4180 *Mya*), the same for the CBM49/ CBM49-like land plants was the presence of a distinct and delineable CBM49 in the ancestral bryophytes and tracheophytes coupled with the assumption that the parent of class C vascular land plants (≈201 − 241 *Mya*) were likely to possess the same architecture (Table 2; Additional file 5: Table S1 and Additional file 8: Table S2) [18, 19].

**Table 2:** Parameters utilized for Bayesian inference of evolution of the GH9 and CBM49 domains

Site	Model: Gamma
	Subsitution rate = 1.0
	Substitution model: WAG/ JTT
	Gamma category count = 5
	Shape: 1.537/ 0.813
	Proportion invariant: NA/ 0.027
Clock	Model: Relaxed clock Log Normal
	Number of Discrete Rates = − 1
	Clock rate = 1.0
Calibrated Yule model	Birth rate = 1.0
	Type (Full)
birthRate	Model: Gamma
	Initial = 1.0 [−∞, ∞]
	*α* = 1.0*E* − 03 *β* = 1.0*E* + 03
	Mode:= Shape Scale
	Offset = 0.0
gammaShape	Model: Gamma
	Initial = 1.0 [−∞, ∞] *α* = 1.0*E* − 03 *β* = 1.0*E* + 03
	Mode: Shape Scale
	Offset = 0.0
Population mean	Model: Exponential
	Initial = 1.0 [−∞, ∞] *μ* = 10.0
	Offset = 0.0
Uncorrelated relaxed local clock mean	Model: Exponential Initial = 1.0 [−∞, ∞] *μ* = 10.0 Offset = 0.0
Uncorrelated relaxed local clock standard deviation	Model: Exponential
	Initial = 1.0 [−∞, ∞] *σ* = 0.3337
	Offset = 0.0
Root	Parent of: Bacteria/ Vascular class C land plants
	Monophyletic
	Model: Log Normal
	*μ* = 8.2/ 5.41 *σ* = 0.07/ 0.055
	Offset = 0.0
	2.5% Quantile = 3170/ 201Mya
	97.5% Quantile = 4180/ 249Mya
Markov chain monte carlo	Chain length = 14,917,000 / 16,120,000
	Pre Burnin = 4,200,000/ 2,130,000
	Recording interval = 1000

*Abbreviations*: *GH9* Glycoside hydrolase 9, *CBM49* Carbohydrate binding module 49, *WAG* Whelan and Goldman, *JTT* Jones, Taylor, and Thornton

**Table 3:** Taxonomic distribution of bacteria in datasets

	Dataset	*n*_1_	*n*_2_
	Number of sequences	116	64
1.	Firmicutes	65	44
	Clostridiales	49	40
	Bacillales	15	4
	Selenomonadales	1	–
2.	Actinobacteria	20	10
	Micrococcales	3	1
	Streptomycetales	11	6
	Streptosporangiales	2	1
	Micromonosporales	2	–
	Pseudonocardiales	2	2
3.	Proteobacteria	20	5
	Gamma (*γ*)	14	3
	Alpha (*α*)	4	2
	Delta (*δ*)	1	–
	Undefined	1	–
4.	CFB	9	3
5.	Cyanobacteria	1	–
6.	Undefined	1	1

*Abbreviations*: *GH9* Glycoside hydrolase 9, *CFB* Chlorobi, Fibrobacteres, Bacteroidetes

### Pattern analysis and motif approximation of CBM49 in putative GH9 endoglucanases

The boundaries of CBM49 were defined in characterized and putative class C GH9 endoglucanase sequences with single- and multiple-copies of the GH9 domain (*n* = 116) (Additional file 5: Table S1C and Additional file 8: Table S2B) [6–8, 83, 84]. These were then clustered, realigned, and represented using the Clustal Omega and WebLogo servers (https://www.ebi.ac.uk/Tools/msa/clustalo; http://weblogo.berkeley.edu/logo.cgi) with default parameters [90–92]. The refined list of CBM49 sequences in (*n* = 100) class C GH9 endoglucanases were then submitted to the PRATT v 2.1 server (http://web.expasy.org/pratt), and utilized to identify and score suitable domain spanning patterns [93]. A profile of these patterns (*n* = 20) was generated based on the numbers of putative class C enzymes that they were found in, i.e., 5 → 100 (Table 4). This was used to search for sequences with CBM49-like motifs amongst full length GH9 endoglucanase sequences without a delineable CBM49 region, and on the GH9 domain itself and was accomplished using the server ScanProsite (http://prosite.expasy.org/scanprosite) (Additional file 9: Table S5). These datasets (*n*_1*B*_, *n*_1*C*_, *n*_2_, *n*_3_) along with the subset of was used for all further analyses (Tables 4, 5 and 6; Additional file 9: Table S5, Additional file 10: Table S6, Additional file 11: Table S7 and Additional file 12: Table S8, Additional file 13 Text S9, Additional file 16: Text S10, Additional file 14: Text S11 and Additional file 15: Text S12). Alternatively, a Hidden Markov Model or support vector machine (SVM) may have been utilized for this part of the analysis. SVMs, are binary classifiers and incorporate several features of the training sequences to determine presence/ absence in an unknown sequence of interest. Whilst the SVM for the CBM49 could have been easily constructed, its utility in identifying the same in a distantly related sequence is likely to be limited. The HMM, however, for this specific module hand would simply indicate the existence of a similar region above a certain threshold. Since, our requirement mandated features of both these, i.e., presence/ absence of CBM49-like regions in GH9 domain containing endoglucanases across taxa, these predictors of the extrema would not have sufficed.

**Table 4:** Alignment based pattern analysis of CBM49 in putative and characterized class C GH9 endoglucanases

	Motif	Fs	Sm	Rm
1	GPIWGLTK[AS]G[DN]SY[GTV]FP[EST][HW][IL][NS][ST]L[APS][AV]GKS[LM]EFVYIH[AS][AT]S	140.4529	5	2.47E-35
2	GPIWGL[ST][KR]SG[DN]S[FY][AGT][FL]P[EST][HW][ILM]x[ST]Lx[AS]GKSLEFVYIH[AS][AT][ST]	131.0036	10	3.02E-32
3	GPIWGL[NST]x(2)[GP][DENQ]x(2)[AGTV]	75.6634	15	7.32E-04
4	GPIWGL[ST]x(2)[GP][DEN]x(2)[AGTV]x[PV]x(4)[STV]x(3)[GQ]x[GS]xE[FV][NV][FY][IV][HY][ASTV][AQT][GPST]	35.2349	20	5.86E-16
5	GPIWG[LV][NST]x[AST][GP][DENQT]x(2)[AGSTV]	36.2141	25	4.10E-04
6	GPIWG[LV][ANST]x(2)[GP][DENQT]x(2)[AGSTV]	33.2127	30	2.92E-03
7	GPI[WY]G[LV][ANST]x(3)[DENQT]x(2)[ADGSTV]	28.9138	35	1.00E-01
8	GP[IL]WGL[ANST]x(3)[ADEGNQ]	28.034	40	0.16
9	GP[IL]WG[LV][NST]x(3)[DENQT]	27.6347	45	0.14
10	GP[IL]WG[LV][AENST]x(3)[ADEGNQT]	26.4888	50	0.39
11	GP[IL][WY]G[LV][AENST]x(3)[ADEGNQT]	25.6627	55	1.4
12	GP[ILV][WY]G[LV][AENST]	23.5981	61	4.9
13	GP[ILV]xG[LV]	18.3557	65	356
14	G[NPS][IL][WY]G[LV][ANST]	22.9977	70	9.2
15	G[NPS][ILV]WG[LV]	20.977	77	15
16	G[NPS][ILV][WY]G[LV]	20.1508	80	53
17	G[DNPQS][ILV][WY]G[LV]	19.4127	85	83
18	G[DENPQST]x(2)G[LV]	12.9137	90	12,445
19	Gx[ILV][WY]G[LV]	17.5296	98	323
20	Gx(3)G[LV]	11.5238	100	33,184

*Abbreviations*: *Fs* Fitness score, *E* Glutamic acid, *Sm* Number of sequences matched, *Q* Glutamine, *Rm* Estimated number of random matches, S Serine, *A* Alanine, *T* Threonine, *L* Leucine, *C* Cysteine, *M* Methionine, *Y* Tyrosine, *I* Isoleucine, *F* Phenylalanine, *V* Valine, *W* Tryptophan, *G* Glycine, *K* Lysine, *D* Aspartic acid, *H* Histidine, *N* Asparagine, *P* Proline, *R* Arginine, *x* Any amino acid

**Table 5:** Distribution of sequence segments in classes A, B, and C plant GH9 endoglucanases

	MEMSAT-SVM		DAS		PHOBIUS	
	SP	TM	SP	TM	SP	TM
C0 (NN)	0.0000 (0/97)		0.0588 (3/51)		0.0000 (0/100)	
C1 (YY)	0.7525 (73/97)	1.0000 (97/97)	0.8604 (37/43)	0.8958 (43/48)	0.0000 (0/2)	0.0200 (2/100)
C2 (NY)	0.2474 (24/97)		0.1395 (6/43)		1.0000 (2/2)	
B0 (NN)	0.0000 (0/75)		0.0196 (1/51)		0.0533 (4/75)	
B1 (YY)	0.8133 (61/75)	1.0000 (75/75)	0.5600 (28/50)	0.9803 (50/51)	0.3333 (1/3)	0.0422 (3/71)
B2 (NY)	0.1866 (14/75)		0.4400 (22/50)		0.6667 (2/3)	
A0 (NN)	0.0000 (0/22)		0.0454 (1/22)		0.0909 (2/22)	
A1 (YY)	0.0000 (0/22)	1.0000 (22/22)	0.0000 (0/21)	0.9545 (21/22)	0.0000 (0/20)	0.9090 (20/22)
A2 (NY)	1.0000 (22/22)		1.0000 (21/21)		1.0000 (20/20)	

*Abbreviations*: *SVM* Support vector machine, *SP* Signal peptide, *TM* Transmembrane region, *DAS* Density alignment surface, *YY* (*SP*^+^) ∧ (*TM* ∨ *PH* ∨ *RH*)^+^, *NY* (*SP*^−^) ∧ (*TM* ∨ *PH* ∨ *RH*)^+^, *NN* (*SP*^−^) ∧ (*TM* ∨ *PH* ∨ *RH*)^−^

**Table 6:** Salient features of putative GH9 endoglucanase sequences with multiple delineable domains

		GH9	CBM2	CBM3	CBM4_9	CBM10	CBMX_2	CBM49	pattern 20
ALS (*n* = 3)	gi|313241202	Y			Y (cT)				Y
	gi|260808721	Y	Y (nT)						Y
	gi|254553092	Y	Y (nT)						Y
BAC (*n* = 24)	gi|15894203	Y		Y (cT)					Y
	gi|15894200	Y		Y (cT)					Y
	gi|15893851	Y			Y (nT)				Y
	gi|300789210	Y	Y (cT)		Y (nT)				Y
	gi|300785821	Y		YY (cT)					Y
	gi|121833	Y		YY (cT)			YY (cT)		Y
	gi|320006799	Y	Y (cT)		Y (nT)				Y
	gi|295094191	Y			Y (nT)				Y
	gi|291544575	Y			Y (nT)				Y
	gi|291543938	Y		Y (cT)					Y
	gi|34811382	Y		Y (cT)					Y
	gi|34811081	Y		Y (cT)					Y
	gi|2554767	Y		Y (cT)					Y
	gi|551774	Y		Y (cT)					Y
	gi|311900744	Y	Y (cT)						Y
	gi|311900370	Y	Y (cT)	Y (cT)					Y
	gi|270288703	Y		Y (cT)					Y
	gi|270288702	Y		Y (cT)					Y
	gi|270288700	Y			Y (nT)				Y
	gi|270288699	Y		Y (cT)					Y
	gi|39636954	Y		Y (cT)					Y
	gi|6272570	Y			Y (nT)				YYYYY
	gi|237858935	Y	Y (cT)			Y (cT)			YY
	gi|4490766	Y		YY (cT)					YYY
PRS (*n* = 2)	gi|281207043	Y						Y (cT)	
	gi|281207029	Y						Y (cT)	

*Abbreviations*: *ALS* Animals, *BAC* Bacteria, *PRS* Protists, *Y* Present, *nT* N-terminal, *cT* C-terminal, *GH9* Glycoside hydrolase 9, *CBM* Carbohydrate binding module

### Domain analysis of plant GH9 endoglucanases

The above compiled datasets (*n*_1_ − *n*_3_) were meant to offer an insight into the origin and evolution of the GH9-CBM49-like domain across all taxa, the end point being the emergence of plant GH9 endoglucanases (classes A, B, and C) (Additional file 6 Table S3, Additional file 7: Table S4, Additional file 9: Table S5, Additional file 10: Table S6 and Additional file 11: Table S7, Additional file 1: Texts S1, Additional file 4: Texts S2, Additional file 2: Texts S3 and Additional file 3: Texts S4. Since the methods discussed afford compelling evidence of the ancestral nature of class C GH9 endoglucanase sequences, our subsequent analyses (domain frequency) was focussed on establishing potential divergence of class C members and/ or the emergence of classes A and B. Plant GH9 endoglucanase sequences possess a differential distribution of TM, SP, and CBM49 regions. and the frequency of occurrence of these was analysed by directly comparing CBM49 positive class C members (*n*_3*C*_ = *n*_*LPSC*_ = 97) with pattern 20 selected sequences of putative classes A (*n*_3*A*_ = *n*_*LPSA*_ = 22) and B (*n*_3*B*_ = *n*_*LPSB*_ = 75) (Additional file 10: Table S6, Additional file 3: Text S4). Since, the hydrophobic profile of these regions overlap, we utilized data from three algorithms that predict both TM and SP regions to arrive at a consensus. The servers consulted were: MEMSAT-SVM, DAS-TMfilter, and PHOBIUS [94–101] (Additional file 11: Table S7, Additional file 13: Text S9, Additional file 16: Text S10 Additional file 14: Text S11 and Additional file 15: Text S12). The MEMSAT-SVM classifies membrane spanning helical regions in a sequence as strong (TM), weak pore-lining (PH), or re-entrant (RH), i.e., (*TM* ∨ *PH* ∨ *RH*). [94, 100]. The dense alignment surface (DAS-TMfilter) differs from other predictors of transmembrane regions in considering hydrophobic region(s) of a query protein, and mapping the results to known transmembrane regions [95, 96]. PHOBIUS, is a hidden Markov model based delineator of signal peptide regions and uses sub models of the sequences that comprise these regions along with topology information to make predictions [101].

### Algorithm to assess contribution of prediction method to each sub segment

Full length sequences of land plants encompassing the CBM49-pattern 20, i.e., classes A, B, and C (*n*_3_ = (*n*_3*LPSA*_ = *n*_3*A*_) + (*n*_3*LPSB*_ = *n*_3*B*_) + (*n*_3*LPSC*_ = *n*_3*C*_) = 187) were searched for well defined amino acid segments using the aforementioned servers (MEMSAT-SVM, DAS, PHOBIUS). The subset (***NN***) was used to define sequences without delineable TM and SP regions (*NN* = {*C*0, *B*0, *A*0}). The method of choice was determined by rendering the resultant data equivalent and therefore, comparable. The definitions utilized are as under:

$$ {\displaystyle \begin{array}{ccc} TM& :=& Sequences\kern0.5em with\kern0.5em one\  or\ more\kern0.5em predicted\kern0.5em transmembrane\kern0.5em domains\\ {} SP& :=& Sequences\kern0.5em with\kern0.5em one\  or\kern0.5em more\ predicted\kern0.5em signal\kern0.5em peptide\ regions\\ {} PH& :=& Sequences\kern0.5em with\kern0.5em one\  or\ more\kern0.5em predicted\kern0.5em pore\ lining\ helices\\ {} RH& :=& Sequences\kern0.5em with\kern0.5em one\  or\ more\kern0.5em predicted\kern0.5em pore\ lining\ helices\\ {}\boldsymbol{NN}& :=& \left({SP}^{-}\right)\wedge {\left( TM\vee PH\vee RH\right)}^{-}\\ {}\boldsymbol{NY}& :=& \left.\Big({SP}^{-}\right)\wedge {\left( TM\vee PH\vee RH\right)}^{+}\\ {}\boldsymbol{Y}\boldsymbol{Y}& :=& \left({SP}^{+}\right)\wedge {\left( TM\vee PH\vee RH\right)}^{+}\\ {}\boldsymbol{Y}& :=& {\left( TM\vee PH\vee RH\right)}^{+}\end{array}} $$

- **Step 1**:Sequences with negative predictions for both SP and TM regions (*f*(***NN***) ↔ *ℕ*) and {*x*_*i*_ ∈ ***NN ⊂*** *n*_3_ ∣ (*SP*^−^) ∧ (*TM* ∨ *PH* ∨ *RH*)^−^, *i* ∈ *ℕ*), were removed from the computations.
- **Step 2**:The remaining sequences were assessed for the presence of the transmembrane subregions (*f*(***Y***) ↔ *ℕ*) and {*x*_*i*_ ∈ ***Y ⊂*** *n*_3_ ∣ (*TM* ∨ *PH* ∨ *RH*)^+^, *i* ∈ *ℕ*).
- **Step 3**:The data computed in Step 2 was then used to calculate the number of sequences with or without the presence of an associated signal peptide regions (*f*(***NY***) ↔ *ℕ*) and (*f*(***YY***) ↔ *ℕ*). {*x*_*i*_ ∈ ***NY ⊂*** *n*_3_| (*SP*^−^) ∧ (*TM* ∨ *PH* ∨ *RH*)^+^, *i* ∈ *ℕ*} and {*x*_*i*_ ∈ ***YY ⊂*** *n*_3_| (*SP*^+^) ∧ (*TM* ∨ *PH* ∨ *RH*)^+^, *i* ∈ *ℕ*}.
- **Step 4**:Utilize the data from the above to compute a ratio was used to establish equivalence between the predictions, and thereby, a rationale for its subsequent inclusion/ exclusion $$ \left(\raisebox{1ex}{$\mid \boldsymbol{NY}\mid $}\!\left/ \!\raisebox{-1ex}{$\mid \boldsymbol{Y}\mid $}\right.,\raisebox{1ex}{$\mid \boldsymbol{Y}\boldsymbol{Y}\mid $}\!\left/ \!\raisebox{-1ex}{$\mid \boldsymbol{Y}\mid $}\right.\right) $$.

## Results

### Taxonomic distribution of the GH9 domain

The GH9 domain averages ≈448 *aa*, and is present as a single copy in the sequences investigated (*n*_1_ = 607), i.e., bacteria (BAC), land plants (LPS), animals (ALS), fungi (FGI), green algae (GAL), protists (PRS), and archaea (ARC) (Fig. 1c; Additional file 5: Table S1A). Although, the vast majority of sequences selected for this study were putative GH9 endoglucanases, available empirical data (kinetic, transcript data, 3D structure) for many of these taxa were available and included (*n*_*LPS*_ = 26; *n*_*ALS*_ = 1; *n*_*BAC*_ = 11). Whilst, most sequences possessed alignment compatible GH9 domains (*n*_1*A*_ = 601), there were few sequences (*n* = 6) which could not be aligned and were not utilized in the estimation of divergence of GH9 domains across taxa (Additional file 5: Table S1A, Additional file 1: Text S1). The source of error was most likely the archaeal sequence (*Methanohalobium evestigatum*; tr|D7E938). This sequence has a predicted GH9 domain length of 222 aa (*Eval* = 1.2*E* − 08), and sub optimally aligned sequences are likely to have inflated scores in excess of the threshold for inclusion. On the other hand, despite possessing GH9 domains of suitable length, the lower confidence levels of the HMM predictor for *α*-proteobacteria (*Asticcacaulis biprosthecum*; gi|328841530, gi|328840708; *Evals* = 2.20*E* − 17, 8.40*E* − 15), and a member each of the Chlorobi-Fibrobacter-Bacillales (CFB) ancestral phylum (*Bacterioides fluxus* YIT 12057; gi|328530713, gi|328531610; *Evals* = 2.80*E* − 25, 2.40*E* − 23)) and subgroup Bacillales of the Firmicutes (*Listeria innocus*; gi|313621564; *Eval* = 1.50*E* − 20) were probable confounders for the alignment mismatch (Additional file 5: Table S1A). The bacterial subgroup comprised Gram negative (proteobacteria) and Gram positive organisms (members of CFB phylum, cyanobacteria, firmicutes, and bacillales) (Table 3, Fig. 1c). However, multiple distinct representations of the GH9 domain in one protein are not uncommon, and are present as two or four (*Saccoglossus kowalevskii*; gi|291236258) copies (*n* = 16; *n*_*ALS*_ = 7, *n*_*BAC*_ = 2, *n*_*LPS*_ = 7) (Additional file 5: Table S1B). Additionally, we observed the concomitant presence of heterogenous Glycoside hydrolase domains in some bacterial species (*n*_*BAC*_ = 4), which included *Caldocellum saccharolyticum* (*gi*| 1708078; *GH*9, *GH*48), *Ruminococcus champanellensis* (*gi*| 291543673; *GH*9, *GH*16), *Ruminioclostridium thermocellum* (*gi*| 1663519; *GH*9, *GH*44), and *Caldicellulosiruptor* spp. (*gi*| 12743885; *GH*9, *GH*44) (Additional file 5: Table S1C). Interestingly, despite being classified as GH9 members, only the anaerobic methanogen (*Methanohalobium evestigatum*; tr|D7E938) of the archaea subgroup Euryarchaeota possessed the requisite GH9 domain (Additional file 5: Table S1D).

### Evolution and emergence of the GH9 and CBMs in plant and non plant taxa

**Fig. 2:**
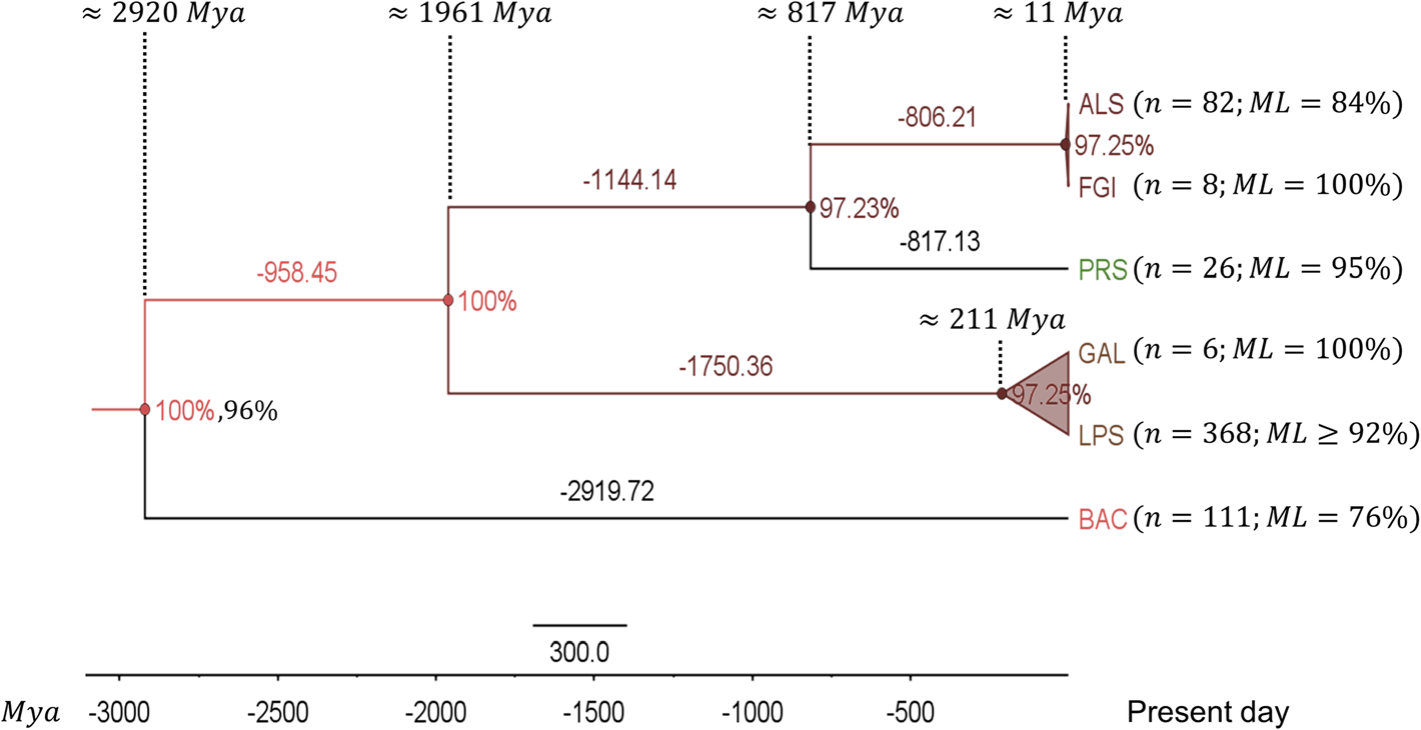
Evolution of GH9 domain. A Bayesian inference (BI) dated tree was estimated (maximum clade credibility) from the computed tree population (*n* = 4476; *burn* − *in* = 70%) using the WAG amino acid substitution model and parent of the clade of bacteria as the root. Whilst, node ages (= node height = branch time of the longest diverging taxa) and branch times are in *Mya*, support for branch points are indicated by the posterior probabilities (*PP*%) and bootstrap values (*n* = 1000; *ML*%), i.e., *support* = *PP* % , *ML*%. The root for this tree was the parent of bacteria (3170 − 4180 *Mya*).The log likelihood for this tree was (≈ − 0.0838233). Abbreviations: BI, Bayesian inference; GH9, glycoside hydrolase; *Mya*, millions of years; WAG, Whelan and Goldman

The data suggests that the GH9 domain is conserved across all taxa and a catalytically functional copy may have been present in bacteria (≈3000 *Mya*; *support* = 100%, 96%) (Fig. 2; Additional file 7: Table S4A, Additional file 17: Text S5 and Additional file 18: Text S6). Interestingly, the clades of the land plants and green algae appears to have diverged relatively early and independently of the animals, fungi, and the protists (≈1961 *Mya*; *support* = 100%). Whilst, the GH9 domains of the land plants and green algae continued to evolve for another ≈1750 *Mya* finally diverging from each other relatively recently (≈211 *Mya*; *support* = 97%). In contrast, the protists diverged from animals and fungi (≈817 *Mya*; *support* = 97%), whilst GH9 domains of animals and fungi diverged from each other (≈11 *Mya*; *support* = 97%). A generic timeline for the evolution of the GH9 domain, i.e., *BAC* > *PRS* > {*FGI*, *GAL*, *ALS*, *LPS*}, is perfectly plausible (Fig. 2). We also posited, and thence investigated the contribution of non-GH9 regions (CBM49, linker(s)) to substrate dichotomy (crystalline, amorphous) in plant GH9 endoglucanases. We observed distinct and delineable CBM49s (79 − 84 *aa*; *median* = 81 *aa*) in putative class C GH9 endoglucanase sequences of flowering land plants (*n* = 102) after outlier exclusion (*n* = 2; *Zea mays, GRMZM2G143747_P01; Selaginella. moellendorffii*, *109529*) (Additional file 8: Table S2A and B). The only exceptions were the presence of a single CBM49 (82 *aa*) in the protist, *Polysphondylium pallidum*PN500 (gi|281207043, gi|281207029) (Additional file 5: Table S1A). Remarkably, our results indicate a unique copy of CBM49 in bryophytes (*n* = 4; *Physcomitrella patens*) and tracheophytes (*n* = 3; *S. moellendorffii*) (Additional file 8: Table S2). Analysis of the primary sequences also indicates the presence of one or more linker sequences connecting the GH9 to the CBMs. In CBM49 class C sequences this constitutes a 7–77 AA (*Prunus persica*, *ppa022524m*; *Phaseolus vulgaris*, *Phvul.011G030300.1*) (Additional file 5: Table S1 and Additional file 8: Table S2).

### Characterization, analysis, and assessment of relevance of CBM49-spanning patterns in non-plant taxa

**Fig. 3:**
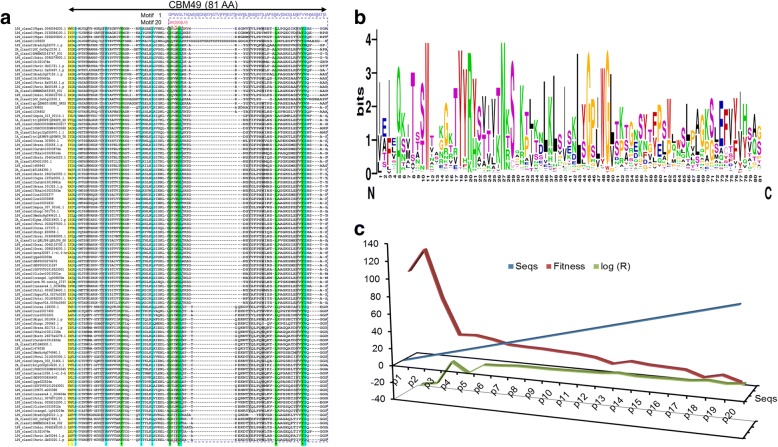
Characterizing the carbohydrate binding module (CBM49). **a** Multiple sequence alignment of the CBM49 in class C GH9 endoglucanases. This region has been highlighted in the presented alignment, and suggests a conservation, in not just the overall structure, but also several key residues (W| F| Y; K| R| N| H| Q). Additionally, the highest (*p*1) and the lowest (*p*20) scoring patterns that approximate CBM49 have been illustrated. The rudimentary *p*20 derived from class C sequences was found in several organisms (*n* = 194), including classes A and B of plant GH9 members, **b** WebLogo of the carbohydrate binding domain 49 of putative class C plant GH9 endoglucanases. Truncated sequences with a well defined 81 AA region corresponding to the CBM49 were utilized to construct this, and **c** Analysis of 20 patterns spanning the CBM49 with number of matched sequences (*Sm*), fitness (*Fs*), and randomly matched sequences (log(*Rm*=R)) as indices. Abbreviations: AA, amino acids; CBM49, carbohydrate binding module; *Fs*, fitness score; GH9, glycoside hydrolase; *Sm*, number of sequences with matches; *Rm*, number of randomly matched sequences

**Fig. 4:**
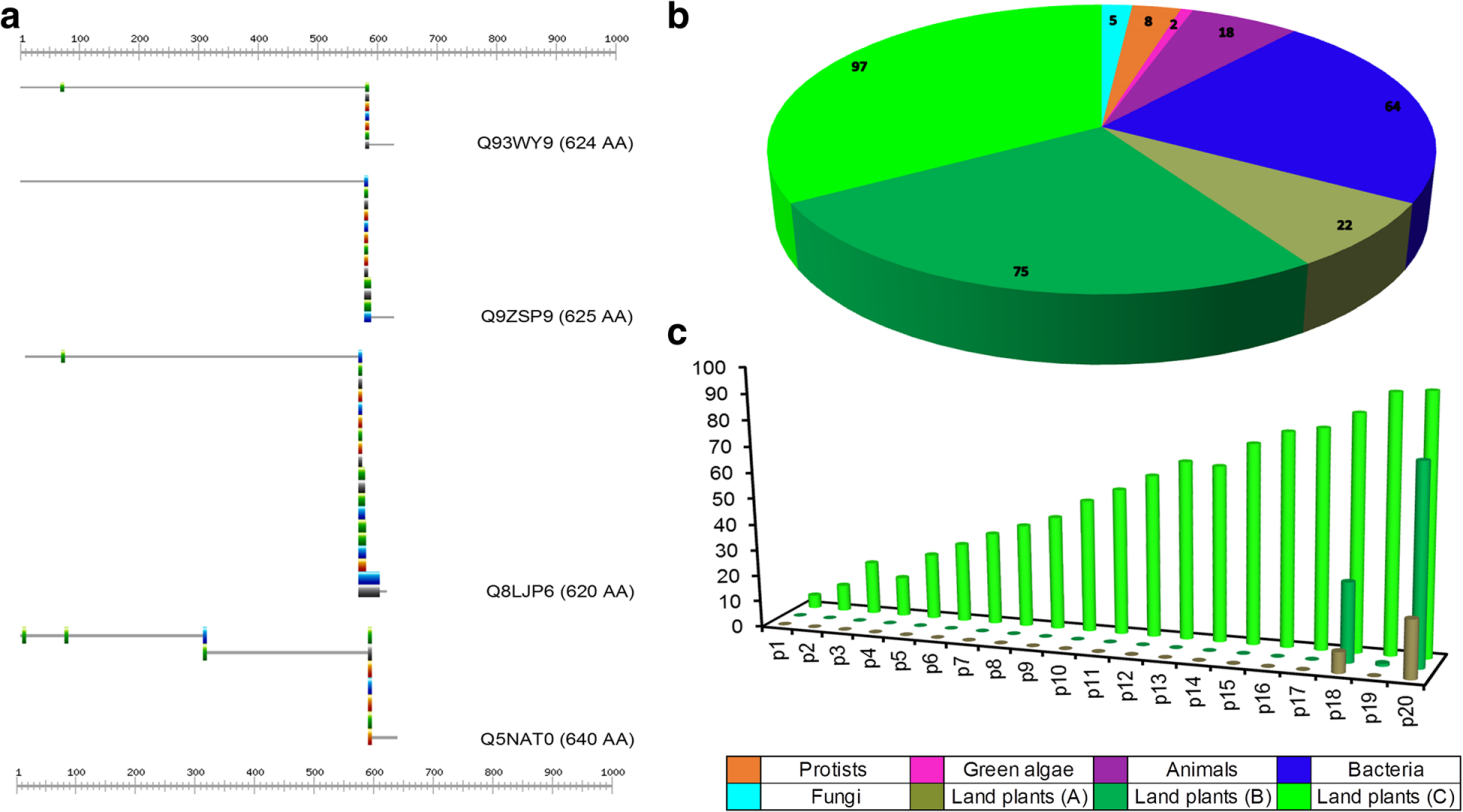
Pattern analysis and major findings in selected plant GH9 endoglucanases. **a** Distribution and presence of high- and low-fitness strength CBM49-spanning patterns (53 'hits' on 4 sequences) in characterized class C enzymes, **b** Taxonomic distribution of the low strength *p*20 (*n*_2_ = 291), and **c** Analysis of the presence of all 20 CBM49-spanning patterns in selected sequences of classes A, B, and C (*n* = 187). Clearly, the ubiquitous presence of *p*20 favours its use as an index of the presence of CBM49 in non class C taxa. The higher strength patterns (*p*1-*p*17) are limited to putative class C GH9 endoglucanases. Abbreviations: CBM49, carbohydrate binding module; GH9, glycoside hydrolase; *p*20, pattern 20

The amino acid profile (*HSC* ≅ 46.2%; *AAA* ≅ 11%; *PUC* ≅ 36%; *PCA* ≅ 4.7%; *PCB* ≅ 13%) of the truncated CBM49 sequences (*n* = 102) suggests a high percentage of amino acids whose side chain functional groups (*PUC* = {−*OH*, −*SH*, −*NH*_2_}), i.e., Serine (S), Threonine (T), Cysteine (C), Tyrosine (Y), Asparagine (N), and Glutamine (Q), could potentially contribute to the catalytic machinery of these putative enzymes (Additional file 8: Table S2C). Interestingly, there was a paucity of the catalytic permissive (*PCA* = {−*COO*^−^}) amino acids (D/E) in the sequences analysed (Fig. 3a and b; Additional file 8: Table S2, Additional file 4: Text S2). Clearly, the restricted taxonomic distribution of CBM49 precludes a direct comparison, thereby justifying our search for patterns that could approximate CBM49 (Fig. 3; Additional file 9: Table S5). These patterns were partitioned into those with low/ high fitness strengths, which was correlated to its compositional complexity (Table 4, Fig. 3c). Since, patterns of reduced complexity are likely to be present in a greater number of sequences, and also possess low fitness (*Fs*) scores (Table 4, Fig. 4c). The *Rm*-value is the expected number of random matches in 100,000 unrelated sequences [102]. For instance, the pattern with the lowest fitness score (*p*20), i.e., Gx(3)G[LV], has the value *Rm* = 33184 (*n* = 100), whilst the same for the high scoring pattern 1 (*p*1) was *Rm* = 2.47*E* − 35 (*n* = 5) (Table 5, Fig. 3c). The presence of these patterns in CBM49-containing characterized class C sequences was confirmed initially, following which, their occurrence in non-class C members was evaluated (Fig. 4a and b).

These data, for full length sequences of putative GH9 endoglucanases without a delineable CBM49 in terms of number of hits and sequences corresponds to: *p*1 − *p*17 (*hits* = 0), *p*18 (*hits* = 93; *sequences* = 81), *p*19 (*hits* = 2; *sequences* = 2), and *p*20 (*hits* = 233; *sequences* = 194) (Additional file 9: Table S5A). The results for all taxa with the GH9 domain: 18 (*hits* = 98; *sequences* = 89), *p*19 (*hits* = 1; *sequences* = 1), and *p*20 (*hits* = 315; *sequences* = 265) (Additional file 9: Table S5B). The low scoring *p*18 (Gx[DENQPST]x(2)G[LV]) and *p*20 (Gx(3)G[LV]) are the only patterns equivalent to the CBM49 which are found in classes A and B along with other taxa, in both full length and GH9 domain sequences (Table 4). The maximal population (≈44 − 48%) and taxa-specific coverage (*ALS*, *BAC*, *FGI*, *PRS*, *LPS*), then justifies the utilization of *p*20 in defining a dataset that could be used to develop an evolutionary trace of putative class C specific endoglucanase activity (Additional file 7: Table S4 and Additional file 9: Table S5, Additional file 13: Text S9, Additional file 16: Text S10, Additional file 14: Text S11 and Additional file 15: Text S12). This combined, i.e., inclusive of class C sequences, dataset (*n*_2_ = 291) of full length putative GH9 endoglucanase sequences then possessed GH9 (*n* = 1) and CBM49-*p*20 (*n* ≥ 1) occurrences, and includes bacteria (*n* = 64), animals (*n* = 18), fungi (*n* = 5), protists (*n* = 8), and green algae (*n* = 2) (Fig. 4b; Additional file 2: Text S3). The distribution of bacteria between the datasets (*n*_1_, *n*_2_) was similar firmicutes (≈56%, ≈69%), actinobacteria (≈17.2%, ≈15.6%), and proteobacteria (≈17.2%, ≈8%) (Table 3). However, the sole archaeal sequence (tr|D7E938) was conspicuous in the absence of the same (Figs. 1c and 4b; Additional file 5: Table S1A). We also observed that while several sequences of land plants, bacteria, and fungi included more than one occurrence of this pattern, green algae, protists, and animals only contained one occurrence of Gx(3)G[LV] (Additional file 9: Table S5A). A search for sequences with pattern 18 (G[DENPQST]x(2)G[LV]), with a marginal increase in fitness strength (| *δ*_*p*20, *p*18_| ≅1.4) eliminated green algae altogether (Table 4; Additional file 9: Table S5). The taxonomic spread for matched occurrences on the GH9 domain (*n*_1_ = 607) with *p*18 (*n*_1*B*_; *n*_*ALS*_ = 3, *n*_*BAC*_ = 34, *n*_*FGI*_ = 3, *n*_*LPSA*_ = 7, *n*_*LPSB*_ = 28, *n*_*LPSC*_ = 14) and *p*20 (*n*_1*C*_; *n*_*ALS*_ = 14, *n*_*BAC*_ = 53, *n*_*FGI*_ = 4, *n*_*PRS*_ = 6, *n*_*LPSA*_ = 14, *n*_*LPSB*_ = 70, *n*_*LPSC*_ = 108), reiterates the generic nature of these patterns (Additional file 9: Table S5B). Interestingly, and in complete contrast is the profile of occurrences of *p*19, which despite its low fitness registers a single hit (class C, *S. moellendorffii*, *109529*).

### Analysis of CBM49 and CBM49-like GH9 endoglucanases of vascular land plants

**Fig. 5:**
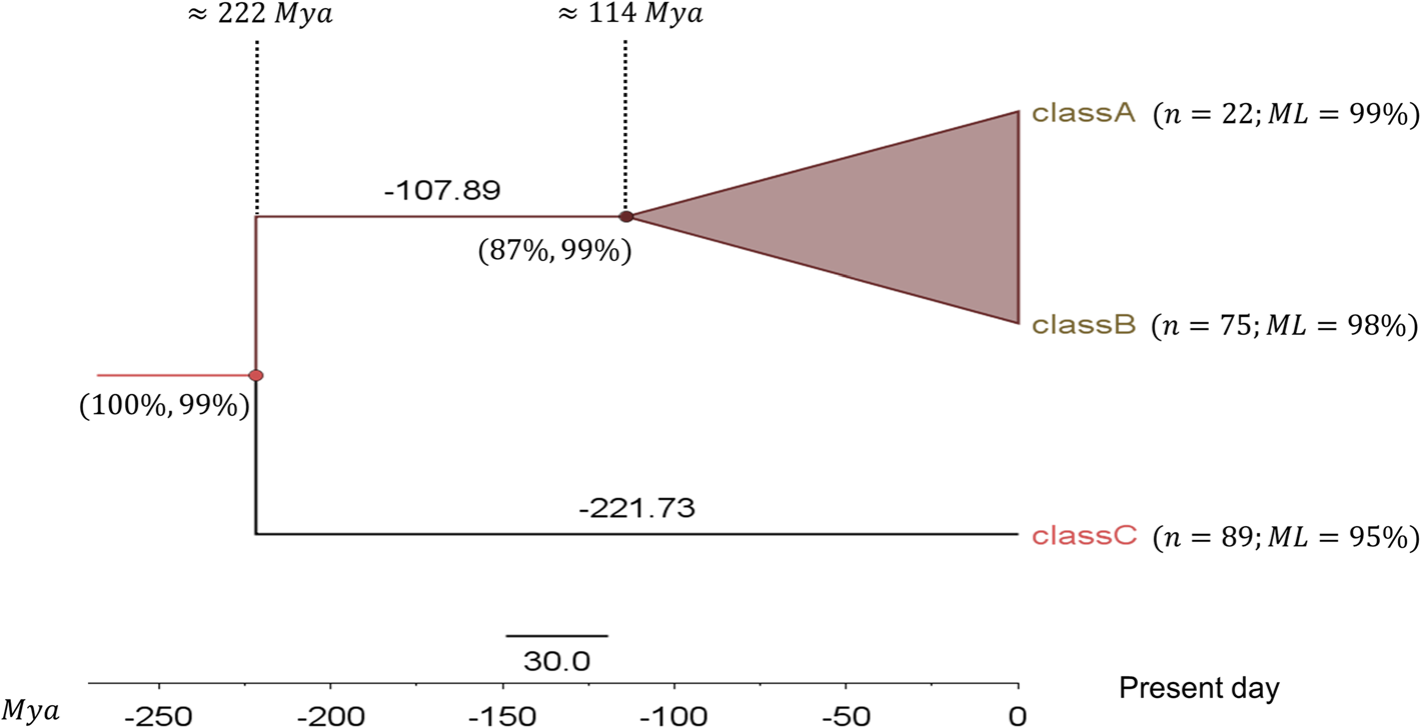
Insights into divergence of plant class C GH9 endoglucanases. A Bayesian inference (BI) dated tree was estimated (maximum clade credibility) from the computed tree population (*n* = 4837; *burn* − *in* = 70%) using the JTT + I + G amino acid substitution model. Whilst, node ages (= node height = branch time of the longest diverging taxa) and branch times are in *Mya*, support for branch points are indicated by the posterior probabilities (*PP*%) and bootstrap values (*n* = 1000; *ML*%), i.e., *support* = *PP%*, *ML*%. The root for this tree was the parent of vascular class C land plants (201 − 249 *Mya*). The log likelihood for this tree was (≈ − 0.1350387). Abbreviations: BI, Bayesian inference; GH9, glycoside hydrolase; I, proportion of invariant sites; G, gamma parameter; *Mya*, millions of years; JTT, Jones, Taylor, and Thornton

In addition to establishing the origins of CBM49, we examined the divergence of putative class C GH9 endoglucanase sequences and the emergence of classes A and B in vascular land plants. To accomplish this a subset of pattern 20 selected GH9 endoglucanase sequences in land plants (*n* = 186; *n*_*LPSA*_ = 22, *n*_*LPSB*_ = 75, *n*_*LPSC*_ = 89) was collated and compared. The node ages and branch times suggest that vascular class C (≈222 *Mya*; *support* = 100%, 99%) GH9 endoglucanases predate members of classes A and B (≈114 *Mya*; *support* = 87%, 99%) (Fig. 5; Additional file 19: Text S7 and Additional file 20: Text S8). The molecular basis of these findings were ascertained by examining CBM49 (class C) and CBM49-like (classes -A and -B) sequences of vascular land plants for the presence of concomitant transmembrane and signal peptide regions (Table 5, Fig. 4a; Additional file 11: Table S7, Additional file 16: Text S10, Additional file 14: Text S11 and Additional file 15: Text S12). The MEMSAT-SVM data clearly suggest that all classes of GH9 endoglucanase sequences possess distinct high- (transmembrane; *n*_*LPSA*_ ≈ 96 % , *n*_*LPSB*_ ≈ 83 % , *n*_*LPSC*_ ≈ 80%) or low- scoring (pore-lining; *n*_*LPSA*_ ≈ 4 % , *n*_*LPSB*_ ≈ 19 % , *n*_*LPSC*_ ≈ 20%) helical regions, with the exception of the class B sequence (*MDP0000199273*), which possessed both classes of helices. Interestingly, a third class (re-entrant helical) was computed in class A members (*n*_*LPSA*_ = 3). When these data were combined, i.e., *TM* ∨ *PH* ∨ *RH*, all classes A, B, and C were shown to possess one or more TM subregions (*n*_*LPSA*_ = *n*_*LPSB*_ = *n*_*LPSC*_ = 100 % ) (Table 5). The same for the DAS-TMfilter (*n*_*LPSA*_ = 95%, *n*_*LPSB*_ = 98%, *n*_*LPSC*_ = 90%), and PHOBIUS (*n*_*LPSA*_ = 91%, *n*_*LPSB*_ = 4%, *n*_*LPSC*_ = 2%) (Table 5). The computations also suggest a bimodal distribution of signal peptide regions ((*SP*^−^) ∧ (*TM* ∨ *PH* ∨ *RH*)^+^ ∶  = ***NY***, (*SP*^+^) ∧ (*TM* ∨ *PH* ∨ *RH*)^+^ ∶  = ***YY***). While, the data for MEMSAT-SVM was (*n*_*LPSB*_ ≅ 80%, *n*_*LPSC*_ = 75%; ***YY***), the same for the DAS-TMfilter was (*n*_*LPSB*_ ≅ 56%, *n*_*LPSC*_ = 86%; *YY*). In contrast, the data from PHOBIUS differed considerably (*n*_*LPSB*_ ≅ 33.3%, *n*_*LPSC*_ = 0; ***YY***), and was applicable to only 3 sequences. The was primarily due the almost complete absence of TM (*n*_*LPSB*_ = 4%, *n*_*LPSC*_ = 2%), or conversely the overwhelming presence of signal peptide regions in classes B and C (*n*_*LPSB*_ ≅ 96%, *n*_*LPSC*_ ≅ 98%) enzymes (Table 5; Additional file 11: Table S7, Additional file 16: Text S10 and Additional file 15: Text S12). However, as discussed vide supra, the corresponding results for the presence of the *TM* ∨ *PH* ∨ *RH* regions in class A GH9 endoglucanases predicted by MEMSAT-SVM (*n*_*LPSA*_ = 100%), DAS-TMfilter (*n*_*LPSA*_ = 95%), and PHOBIUS (*n*_*LPSA*_ = 91%) was almost identical (Table 5). Additionally, whilst, the results from DAS-TMfilter were similar to MEMSAT-SVM, its coverage of classes B (*n*_*LPSB*_ = 67%) and C (*n*_*LPSC*_ = 51%) was suboptimal. The MEMSAT-SVM data, therefore was deemed most appropriate for predicting the molecular events that may have occurred during the evolution of plant GH9 endoglucanases (Table 5; Additional file 11: Table S7, Additional file 15: Text S12).

## Discussion

### Evolutionary significance of crystalline cellulose digesting non plant GH9 endoglucanases

Our results, on the evolution of the GH9 and CBM49 regions suggest a pyramidal model with vertical gene transfer and progressive evolution (loss or modification of function) as a plausible explanation for the emergence, occurrence, and divergence of GH9 endoglucanase activity (≈3000 *Mya*) (Figs. 2 and 5) [15–28, 32–34]. Conversely, since crystalline cellulose is the preferred substrate, this also implies a conserved active site architecture of the encoded protein and a correspondingly similar reaction chemistry in non-plant taxa and land plants with putative class C GH9 endoglucanase activity (Tables 1 and 4, Fig. 4a; Additional file 5: Tables S1 and Additional file 8: Table S2) [7, 46, 47].

The structure of crystalline cellulose renders it resistant to alterations in temperature, salt, pH of the surrounding environment, clearly a desirable trait in archaea (methanogens) and bacteria (halophiles, thermophiles) which inhabit extreme environments such as hot springs and the oral and gastrointestinal microbiomes of several animals. Here, perhaps, the role of GH9 endoglucanases could be critical in remodelling the cell membranes, thereby maintaining intracellular homeostasis [47, 49]. Additionally, crystalline cellulose is inert, compact, and insoluble in aqueous and several organic solvents. These physicochemical properties would imply that spores and seeds made predominantly of this polymer would be resistant to dessication and stressors such as weather fluctuations [14, 41, 43]. Clearly, protists (*Dictyostelium-* and *Polysphondylium-spp.*) and gram positive bacteria may have utilized GH9 endoglucanses to regulate the processes of sporulation, dissemination, and effective germination [14, 41, 43]. The lipopolysaccharides (complexes of crystalline cellulose with lipids) synthesized by gram negative bacteria (proteobacteria, actinobacteria) and fungi, too, could aid protection of the organism from host immune systems (phagocytosis) while concomitantly establishing an infection (*Cryptococcus neoformans, Pseudomonas* spp., *Vibrio* spp.) or infestation in developing protists and marine invertebrates [9–14, 41, 43, 48, 102–104]. Reciprocally, an interesting utility of GH9 endoglucanases is to facilitate the symbiotic/ parasitic association between some fungi and bacteria of animal and plants hosts (macrophages, leguminous nodules of the rhizomes) by digesting the crystalline cellulose of the host. Thus, bacteria/ fungi could secrete these enzymes and/ or in association with the cellulosome could digest the cellulose and hemicellulose in root hairs and wood to extract/ exchange nutrients (*Laccaria bicolor, Sporisorium reilianum, Phanerochaete chrysosporium*) [42, 44, 53, 54, 105–109]. Although cellulose is unequivocally inert, reports of its potential to stimulate an immune response in the host are not unknown. In fact, specialized cells in the tunics of marine vertebrates (*O. dioica, S. kowalevskii, and C. intestinalis*) might function as primitive phagocytes that could detect the presence of crystalline cellulose (potential pathogen, index of nutritional status) and could moderate a suitable response (adhesion to the substratum, infection by marine microbes). The ability to utilize the nutritionally superior crystalline cellulose may be an important consideration, *albeit*, indirect for the dominant global presence of arthropods including insects (*Apis mellifera*, *Camponotus floridanus*, *Nasonia vitripennis, Nasutitermes Takasagoensis*), crustaceans (*Daphnia pulex*), and segmented worms (Additional file 5: Table S1A, Additional file 1: Text S1) [15, 50, 51, 108–114]. Since, GH9 endoglucanase producing bacteria populate the microbiomes of these animals, they are able to extract glucose from diverse substrates (wood, chitoligosaccharides) and can subsist in several seemingly inhospitable environments. Additionally, and in comparison to the kingdom specific analysis (bacteria, fungi, land plants, animals) with corresponding multiple trees by previous investigators, we were able to generate a unified time tree of over 600 GH9 domain sequences spread over every major taxa (*n*_*BAC*_ ≈ 6.5*X*, *n*_*ALS*_ ≈ 3.4*X*, *n*_*FGI*_ ≈ 1.6*X*, *n*_*LPS*_ ≈ 4.8*X*), and include green algae and protists [55].

### Rationale and relevance of a multimodal approach to approximating the CBM49

As discussed vide supra, the carbohydrate binding module CBM49 is unique to class C members of land plants (Fig. 3; Additional file 8: Table S2 and Additional file 4: Text S2). Our data suggests that homologous CBMs (*GH*9 ∧ (*CBMx*)_*y*_| *x* ∈ {2, 3, 4,10,49, *X*}, *y* = {1, 2}) distributed across the length of the protein might contribute to catalysis of crystalline cellulose in bacteria (*n* = 37), animals (*n* = 18), and protists (*n* = 2) (Table 6; Additional file 12: Table S8). The data from the SMART server also indicated the presence of several low complexity regions both, in full length and truncated (GH9 domains) sequences. This coupled with the sparse CBM data (<10%), prompted us to search for CBM49 spanning patterns amongst putative non class C GH9 endoglucanase sequences, reasoning that patterns with low fitness scores might constitute a superior index of approximating the CBM49. In our analysis the CBM49-approximating and low scoring *p*18 (Gx[DENQPST]x(2)G[LV]), *p*19 (Gx[ILV][WY]G[LV]), and *p*20 (Gx(3)G[LV]), possessed amino acids that may be both potentially catalytic and/ or facilitatory. Whilst, the bulky side chains of the aromatic amino acids can physically stretch the glycosidic linkage between adjacent *β*(D)-glucopyranose residues and weaken it several fold, amino acids with side chain functional groups (−*OH*, −*NH*_2_, −*SH*), can effect electron-proton transfers and are critical components of the catalytic machinery of any enzyme [62–64, 115]. The concomitant occurrence of these residues with the GH9, i.e., (*GH*9 ∧ *p*18) ∨ (*GH*9 ∧ *p*20), could function as an index of CBM49-presence on the GH9 domain in sequences of non class C taxa and can then be utilized to trace the origins of CBM49. The biological relevance of this approach may be gleaned by examining the correlation between the presence of aromatic amino acids which are known to influence catalysis of crystalline cellulose and the 'hits' or 'occurrences' of low strength patterns in non class C enzymes (Table 4; Additional file 9: Table S5) [62–78]. Whilst, the complete absence of aromatic acids could be responsible for the generic distribution of *p*18 and *p*20 (93 ≤ *n*_*Hits*_ ≤ 230, *full length*; 98 ≤ *n*_*Hits*_ ≤ 315; *GH*9 *domain*), the incorporation of a single residue W/Y into *p*19 results in a significant reduction in its occurrence in non class C members (*n*_*Hits*_ = 2, *full length*; *n*_*Hits*_ = 1; *GH*9 *domain*) (Table 4, Fig. 4b; Additional file 9: Table S5).

### Evolution of the CBM49 encompassing class C GH9 endoglucanases

The identification of the CBM49 as the facilitator of crystalline cellulose digestion (class C activity) in a select population of previously annotated GH9 endoglucanases in land plants raises intriguing queries with regards to the origin, subsequent divergence, and physiological relevance of substrate shuffling (amorphous, crystalline) in plant GH9 endoglucanases [6–8, 33, 34]. In the absence of an identifiable CBM49, the analysis of full length putative GH9 endoglucanase sequences with occurrences of *p*20 (low strength generic approximator of CBM49) might constitute a viable approach, and provide insights into the origins and subsequent divergence of CBM49 containing enzymes.

#### Emergence and origin of the CBM49

The influence of non-GH9 regions of the primary sequence on the catalytic spectrum of plant GH9 endoglucanases, suggest that these, like the GH9 may have originated in non-plant taxa. These could include the presence of: a) homologous CBMs throughout the length of the protein sequence, and b) delocalized residue- specific activity of the GH9 domain itself. Extensive sequence analysis of full length and GH9 domain sequences of non-plant taxa reveals the presence of several regions of low complexity, along with sparsely present pre-defined CBMs (*n* = 57; ≅9.3%) (Table 6; Additional file 12: Table S8). The numbers notwithstanding, distinct copies of CBM2 (animals, bacteria), CBM3 (bacteria), CBM4 (animals, bacteria), CBM10 (bacteria), CBMX (bacteria), and the CBM49 (protists) itself (*GH*9 ∧ (*CBMx*)_*y*_), have been characterized in literature with the encompassing GH9 endoglucanases exhibiting a clear preference for crystalline cellulose [39–53] (Table 6; Additional file 12: Table S8). Interestingly, the CBMs 2 and 4 of animals and bacteria were present at opposite termini of the GH9 domain. Thus, while CBM4_9 is C-terminal in animals, its position in bacteria is distinctly N-terminal, with the reverse being true for CBM2 (Additional file 12: Table S8). This mobility of CBMs across taxa suggests that either N- or C-terminal positioned CBMs could have functioned as precursors of CBM49. The length of the linker sequences exhibited considerably greater variation in non-plant taxa (27 − 230 *aa*) as compared to land plants (7 − 77 *aa*) (Additional file 5: Table S1A,  Additional file 8: Table S2A and Additional file 12: Table S8). In contrast, the low strength CBM49-approximator, i.e., pattern 20, could be mapped directly onto the full length and GH9 domains ( ≅ 50%). In the presence of key aromatic and/ or polar uncharged amino acids this mapping could also confer competency to digest crystalline cellulose. Whilst, the exact origin of the CBM49 remains speculative, our results when combined indicate a distinct probability (>0.00) that a double ((*GH*9 ∧ (*CBMx*)_*y*_) = {0.093} ∨ (*GH*9 ∧ *p*20) = {0.44,0.48}) or triple event ((*GH*9 ∧ (*CBMx*)_*y*_ ∧ *p*20) = {0.041,0.046}) may have resulted in the emergence of CBM49 in early land plants (Table 6; Additional file 12: Table S8).

#### Divergence of class C GH9 endoglucanases

**Fig. 6:**
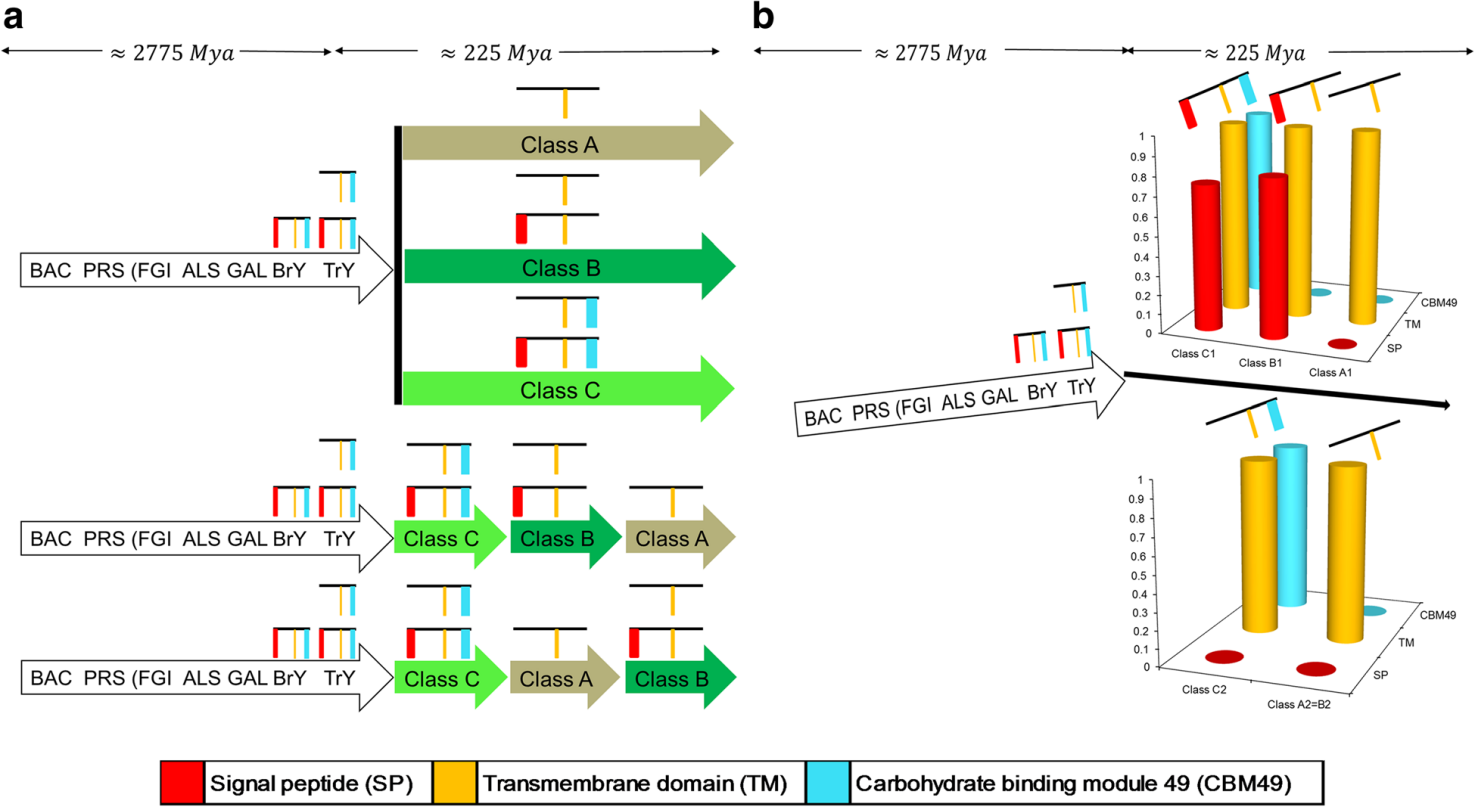
Evolution, divergence, and emergence of plant class C GH9 endoglucanases. **a** Evolutionary theories for the emergence and divergence of classes A, B, and C plant GH9 endoglucanases. The major considerations in proposing these were data gleaned from the time trees, and analysis of the sequences for the presence and/ or absence the transmembrane, signal peptides, and the CBM49 itself. **b** Phylogenetic and bioinformatics analysis of full length sequences of classes A, B, and C plants endoglucanases with one or more occurrences of *p*20. CBM49 attributable class C activity along with a GH9 domain was present in early land plants bryophytes (avascular) and tracheophytes (vascular), and suggests the presence of two class C populations which may have diverged so as to result in the newer classes A and B. Abbreviations: CBM49, carbohydrate binding module; GH9, glycoside hydrolase; *p*20, pattern 20

The interdomain linker, a common feature between the GH9 and CBMs is, surprisingly stable and seems to have remained as such for ≅450 − 480 *Mya*. Whilst, the evidence for the ancestral role of class C members of vascular land plant GH9 endoglucanases is fairly unequivocal, a clear insight into the downstream molecular events that may have occurred in their transformation to classes A and B is debatable (Figs. 5 and 6). Here too, we posited that vertical gene loss of class C GH9 endoglucanase sequences was operative and could result in the emergence of classes A (A1) and B (B1, B2) (Table 5, Fig. 6; Additional file 11: Table S7, Additional file 13: Texts S9, Additional file 16: Text S10, Additional file 14: Text S11 and Additional file 15: Text S12). The extensive computational analysis conducted in this work suggests that classes B (B1, B2) and C (C1, C2) could be considered a union of two distinct groups each, a partitioning that is based on the presence or absence of a signal peptide region (Table 5, Fig. 6; Additional file 11: Table S7, Additional file 13: Texts S9, Additional file 16: Text S10, Additional file 14: Text S11 and Additional file 15: Text S12). The first model purports that the last common ancestor (LCA) of vascular plant GH9 endoglucanases were class C-like enzymes in bryophytes and early tracheophytes. Subsequent losses, in parallel of the CBM49 could have resulted in the appearance of modern vascular equivalents (Figs. 5 and 6). This model also offers an explanation to the fewer numbers of class C members frequently observed by investigators, despite contrasting bioinformatics evidence [14, 58–60]. Indeed, this may be the route of choice for the emergence of class C (≈222 *Mya*; *support* = 100%, 99%) and classes A and B (≈114 *Mya*; *support* = 87%, 99%) (Figs. 5 and 6). Clearly, this model would mandate the presence of distinct subpopulations of the LCA, i.e., CBM49 with either TM or SP regions. Alternatively, class C GH9 endoglucanases of land plants may have been the first to emerge after the tracheophytes, whilst classes A and B evolved from them by the progressive loss of the signal peptide. This route, too, seems perfectly plausible given the presence of two distinct sub populations of class C GH9 endoglucanases (C1, C2), with each diverging secondary to the loss of the CBM49 subregion (*class C*2 → *class A*1 ≈ *class B*2; *n*_1*A*_) and the considerable earlier divergence of class C vascular plants (Table 5, Figs. 5 and 6). Since, classes A and B, in vascular land plants could be originate in parallel and directly from their class C counterparts, the fewer numbers observed could simply mean fewer original class C members left as compared to class B GH9 endoglucanases. A third scenario, could be the origin of later members sequentially, i.e., *class C* → *class A* → *class B* or *class C* → *class B* → *class A* (Fig. 5). Phylogenetic and sequence analysis of this dataset (*n*_3_) suggests that the most probable routes was *class C*1 → *class B*1 → *class A*1 and/ or (*class C*2 → *class A*1 ≈ *class B*2; *n*_1*A*_) (Fig. 6).

### Class C GH9 enzymes, last common ancestor of plant GH9 endoglucanases

Physiologically, the development of an intact vascular system could have brought about a paradigm shift in not just the utilization of extant endoglucanase activity, but also in the nature of cellulose itself. The introduction and persistence of water molecules between the microfibrils of cellulose could have resulted in competition for hydrogen bonds with water rather than other fibrils of cellulose. These events could have been complemented by the late emergence of the crystalline cellulose (*I*_*α*_, *I*_*β*_) editing subclass A GH9 endoglucanases, and could have shifted the reaction equilibria towards the right, i.e., synthesis of amorphous cellulose (*I*_*α*_*am*, *I*_*β*_*am*) [10]. These reactions can be depicted as:

The proliferation of amorphous regions would have rendered cellulose accessible and amenable to enzymatic conversion with lesser stringency. Evolutionarily, this means that the CBM49 in land plants (avascular and early vascular) despite its ancestral origins may no longer be necessary for cellulose metabolism. This in turn may have initiated a series of molecular events in extant class C endoglucanase sequences of late tracheophytes such as *S. moellendorffii*, and may have culminated in the divergence and subsequent appearance of late vascular GH9 endoglucanases of class C (Table 5, Fig. 6) [62–64]. The presence of the linker region too, may have facilitated the progressive loss of CBM49 and its progressive transformation into classes A and B over ≅114 *Mya* (Fig. 5). Since, the modified chemistry and quantity of cellulose made it amenable to rapid digestion, enzymes of classes A and B were more suited to digesting the now abundant amorphous regions of cellulose, and could utilize it as a source of carbon, as well as remodel it to effect growth, development, flowering, and germination [16, 58]. Whilst the presence of crystalline cellulose in the stems of cereal crops (*Hordeum vulgare*, *Brachypodium distachyon*, *O. sativa*) facilitates growth and cultivation, its secretion in the mucilage from the epidermal cells of differentiating eudicot seeds is a critical event in germination [58, 60, 116–118]. The recent divergence of land plant GH9 endoglucanases into monocots such as the cereals (*O. sativa*, *B. distachyon*, *Panicum virgatum*) and the asterid subdivision of the eudicots (*S. tuberosum, S. lycopersicum* and *N. tabacum)* is consistent in all classes and in both datasets (*n*_1*A*_, *n*_3_) (Table 1). These could reflect a modification of the culinary habits of a developing civilization with a desire for bulk and storage foods (Table 4). Here, too the in situ digestion of crystalline cellulose by class C enzymes or its conversion to amorphous forms thereof, could proceed unhindered. The continuing molecular evolution of classes A and B enzymes also suggests a versatile and adaptive mechanism of action perhaps in tandem with the emergence of novel pathophysiological stimuli. The existence of high levels of mRNA of putative class C members observed from the internode regions (high cellulose content) of the developing stems of *O. sativa* and *A. thaliana*, suggest that these enzymes could still be of benefit to modern land plants, as they could direct the higher affinity classes A and B enzymes to regions of growth and development, where the concentrations of cellulose would be much lower [16, 58, 60, 116–118]. The CBM49 of class C plant GH9 endoglucanases could also function as a gene/ protein repository for newly emerging functions, thus justifying their title as living fossils of the plant world.

## Conclusions

Our work when coupled with extant data on class C plant GH9 endoglucanases suggests that these enzymes are ancestral to classes A and B of this family. Plant GH9 endoglucanases are able to digest crystalline cellulose (class C activity) in a manner reminiscent of catalysis by bacteria, animals, protists, fungi, and archaea. Our work here suggests that the GH9 domain is relatively well conserved across taxa. We also present plausible phylogenetic time lines coupled with bioinformatics evidence that favour a vertical mode of gene evolution that may have contributed to the origin and emergence of the CBM49 between the GH9 endoglucanases of plants and non plant taxa, as well as its subsequent divergence (tracheophytes and the vascular land plants of classes A, B, and C). Finally, we review the computational evidence in context of likely physiological events that may have occurred during their divergence and evolution.

## Additional files

Additional file 1: **Text S1.** Sequences of GH9 in all taxa (fasta). (FASTA 283 kb)

Additional file 2: **Text S3.** Sequences with pattern 20 across all taxa (fasta). (FASTA 197 kb)

Additional file 3: **Text S4.** Sequences of land plants (CBM49, pattern 20; fasta). (FASTA 114 kb)

Additional file 4: **Text S2.** Sequences of CBM49 in predicted class C land plants (fasta). (FASTA 11 kb)

Additional file 5: **Table S1.** GH9 domain based classification of taxa. (XLSX 54 kb)

Additional file 6: **Table S3.** Maximum likelihood based evaluation of amino acid substitution models. (XLSX 30 kb)

Additional file 7: **Table S4.** Posterior probabilities for parameters utilized to date GH9/ CBM49 evolution across taxa. (XLSX 15 kb)

Additional file 8: **Table S2.** CBM49 based classification of land plants. (XLSX 22 kb)

Additional file 9: **Table S5.** Distribution of low strength patterns in non class C taxa. (XLSX 41 kb)

Additional file 10: **Table S6.** Distribution of low strength patterns in non class C land plants. (XLSX 19 kb)

Additional file 11: **Table S7.** Distribution of TM, SP, and CBM49 in land plants. (XLSX 22 kb)

Additional file 12: **Table S8.** Distribution of CBMs and low strength patterns in taxa. (XLSX 18 kb)

Additional file 13 **Text S9.** Distribution of low strength patterns in land plants. (TXT 84 kb)

Additional file 14: **Text S11.** Distribution of TM and SP in land plants (DAS-TMfilter). (TXT 36 kb)

Additional file 15: **Text S12.** Distribution of TM and SP in land plants (MEMSAT-SVM). (ZIP 2102 kb)

Additional file 16: **Text S10.** Distribution of TM and SP in land plants (PHOBIUS). (TXT 9 kb)

Additional file 17: **Text S5.** Maximum clade credibility tree to assess evolution of the GH9 domain. (TXT 5 kb)

Additional file 18: **Text S6.** Maximum likelihood estimate of branching times of GH9 evolution with bootstrapping. (PDF 49 kb)

Additional file 19: **Text S7.** Maximum clade credibility tree to assess divergence of the CBM49 in land plants. (TXT 2 kb)

Additional file 20: **Text S8.** Maximum likelihood estimate of branching times of CBM49 in land plants with bootstrapping. (PDF 10 kb)

## Abbreviations

AAA: Aromatic amino acids
ALS: Animals
ANN: Artificial neural network
BAC: Bacteria
BEAST: Bayesian evolutionary analysis by sampling trees
BRY: Bryophytes
CAZy: Carbohydrate active enzymes
CBM: Carbohydrate binding module
DAS-TMfilter: Density alignment server
dbCAN: Database of carbohydrate enzymes annotated
EC: Enzyme commission
FGI: Fungi
GAL: Green algae
GH: Glycoside hydrolase
HMM: Hidden markov model
HSC: Hydrophobic side chains
*I*_*α’*_*I*_*β’*_: Crystalline cellulose
*I*_*α*_*am,I*_*β*_*am,*: Amorphous cellulose
LCA: Last common ancestor
LPS: Land plants
MEGA: Molecular evolutionary genetic analysis
MSA: Multiple sequence alignment
*Mya*: Millions of years
PCA: Polar charged acidic
PCB: Polar charged basic
PIR: Protein information server
PRATT: Pattern analysis
PRS: Protists
PUC: Polar uncharged
SMART: Simple modular architecture research tool
SP: Signal peptide
SVM: Support vector machine
TM: Trans-membrane
TRY: Tracheophytes
