# Supplementary figures and images for: Origin, evolution, and divergence of plant class C GH9 endoglucanases
Source: BMC Evol Biol. 2018 May 30;18:79. doi: 10.1186/s12862-018-1185-2 (PMC5977491; doi:10.1186/s12862-018-1185-2)

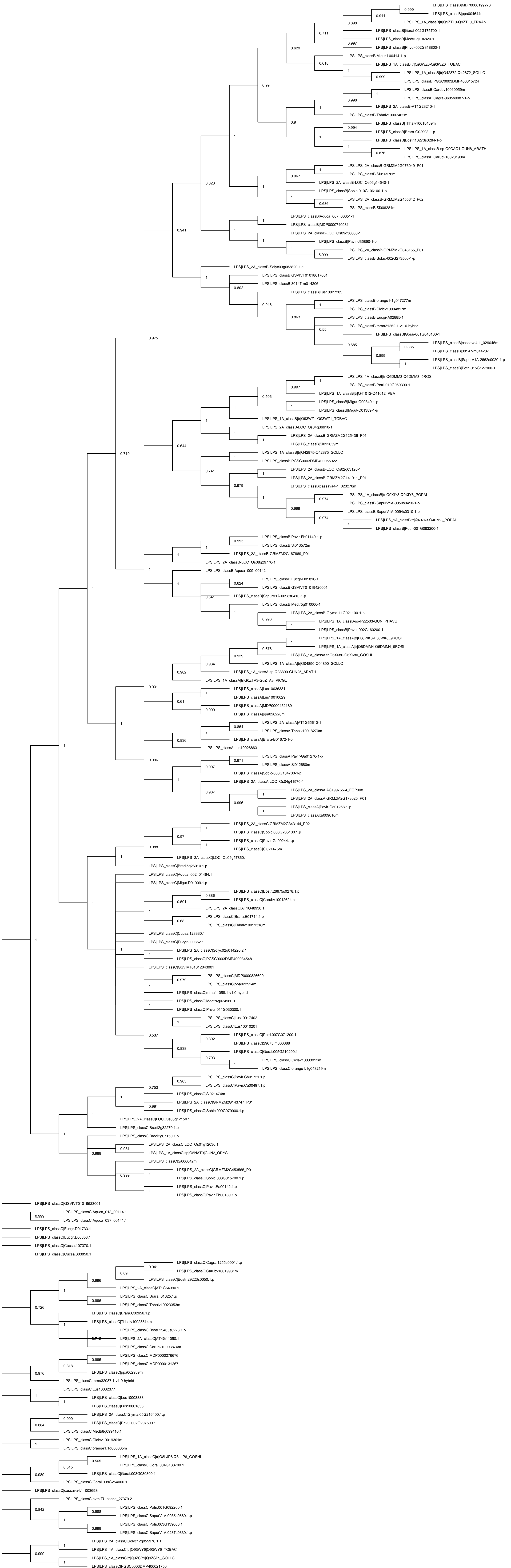

Supplement: Supplementary file 20 — Text S8. Maximum likelihood estimate of branching times of CBM49 in land plants with bootstrapping. (PDF 10 kb) [file 12862_2018_1185_MOESM20_ESM.pdf]
