# Supplementary material for: Origin, evolution, and divergence of plant class C GH9 endoglucanases
Source: BMC Evol Biol. 2018 May 30;18:79. doi: 10.1186/s12862-018-1185-2 (PMC5977491; doi:10.1186/s12862-018-1185-2)
Supplement: Supplementary file 15 — Text S12. Distribution of TM and SP in land plants (MEMSAT-SVM). (ZIP 2102 kb) [file 12862_2018_1185_MOESM15_ESM.zip › Supplementary Text S14/seq_12_classC.zip!/295d4612-0eb6-11e7-a113-00163e110593.>2A_classC|GRMZM2G453565_P01.psipred.pdf]

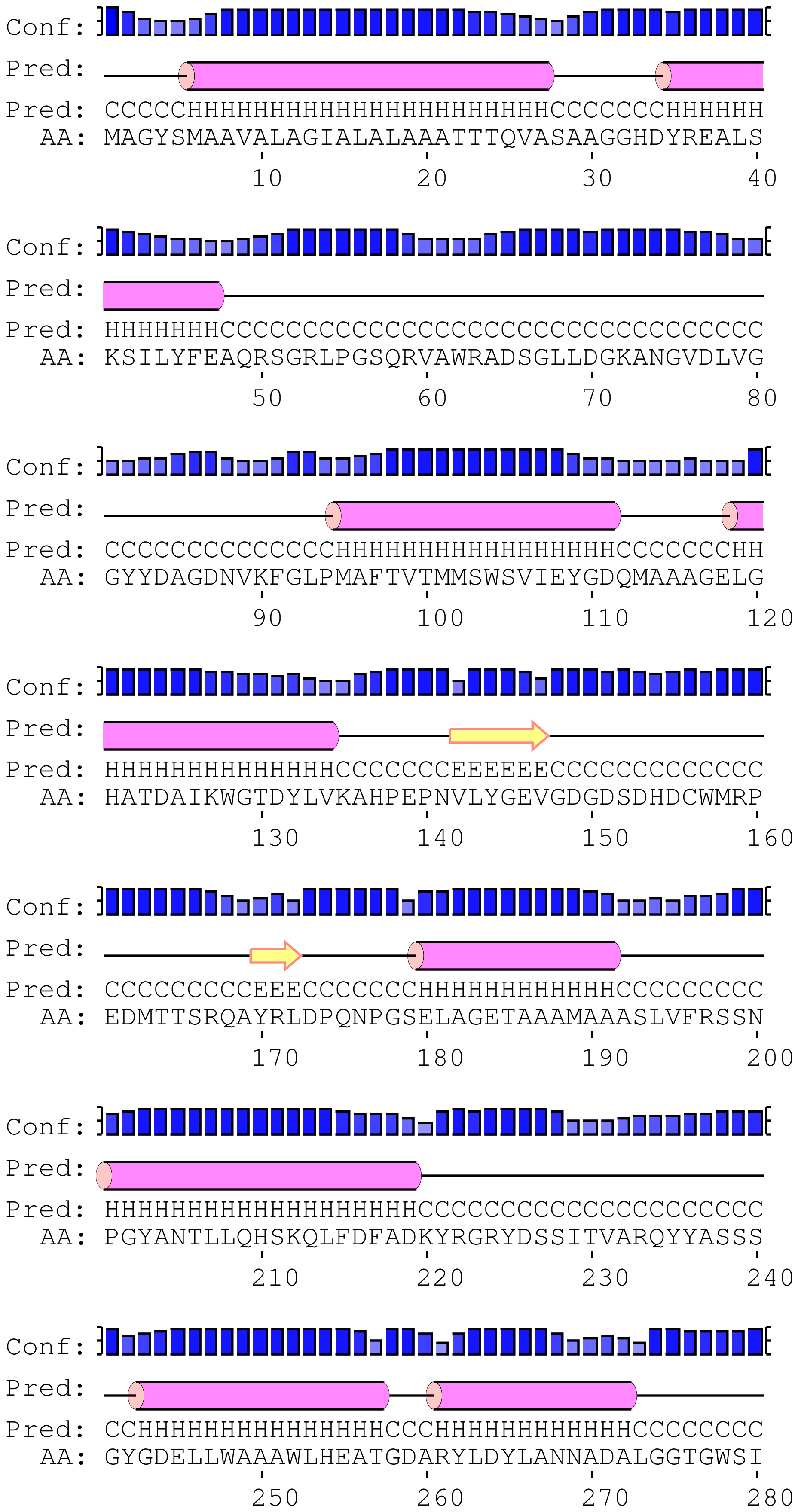
Conf:
Pred:
Pred: CCCCCHHHHHHHHHHHHHHHHHHHHHHCCCCCCCHHHHHH
AA: MAGYSMAAVALAGIALALAAATTTQVASAAGGHDYREALS
10
20
30
40
Conf:
Pred:
Pred: HHHHHHHCCCCCCCCCCCCCCCCCCCCCCCCCCCCCCCCC
AA: KSILYFEAQRSGRLPGSQRVAWRADSGLLDGKANGVDLVG
50
60
70
80
Conf:
Pred:
Pred: CCCCCCCCCCCCCCHHHHHHHHHHHHHHHHHCCCCCCCHH
AA: GYYDAGDNVKFGLPMAFTVTMMSWSVIEYGDQMAAAGELG
90
100
110
120
Conf:
Pred:
Pred: HHHHHHHHHHHHHHCCCCCCCEEEEEECCCCCCCCCCCCC
AA: HATDAIKWGTDYLVKAHPEPNVLYGEVGDGDSDHDCWMRP
130
140
150
160
Conf:
Pred:
Pred: CCCCCCCCCEEECCCCCCCHHHHHHHHHHHHCCCCCCCCC
AA: EDMTTSRQAYRLDPQNPGSELAGETAAAMAAASLVFRSSN
170
180
190
200
Conf:
Pred:
Pred: HHHHHHHHHHHHHHHHHHHHCCCCCCCCCCCCCCCCCCCC
AA: PGYANTLLQHSKQLFDFADKYRGRYDSSITVARQYYASSS
210
220
230
240
Conf:
Pred:
Pred: CCHHHHHHHHHHHHHHHHCCCHHHHHHHHHHHHCCCCCCC
AA: GYGDELLWAAAWLHEATGDARYLDYLANNADALGGTGWSI
250
260
270
280

```
Conf:
Pred:
Pred: CCCCCCCCHHHHHHHHHHHHHHHHCCCCCCCCCHHHHHHH
  AA: NQFGWDVKYPGVQVLAAMTLLRRRGDAGAGAHADVLQRYK
                290       300       310       320

Conf:
Pred:
Pred: HHHHHHHHCCCCCCCCCCEECCCCCEEEECCCCCHHHHHH
  AA: QQADLFACSCLGRGGPNSVRRTPGGMVYHQNWNNVQFVTS
                330       340       350       360

Conf:
Pred:
Pred: HHHHHHHHHHHHHHCCCCEECCCCCCCCHHHHHHHHHHHH
  AA: AAFLLAAYADHLAAAGQGARCPAGGTAQPSELLAFARSQV
                370       380       390       400

Conf:
Pred:
Pred: CHHCCCCCCCEEEECCCCCCCCCCCCCCCCCCCCCCCCCC
  AA: DYILGSNPRATSYMVGYGATYPRQVHHRGASIVSVRANPS
                410       420       430       440

Conf:
Pred:
Pred: CCCCCCCCCCCCCCCCCCCCEEECCCCCCCCCCCCCCCCC
  AA: FVSCQAGYSSWYHRRAANPNLIVGATVGGPDEYDNFADER
                450       460       470       480

Conf:
Pred:
Pred: CCCCCCCCHHHHHHHHHHHHHHHHHHCCCCCCCCCCCCCC
  AA: DNYEQTEATTYNNAPLMGVLARLAAGHGGGGGKFGHHSLA
                490       500       510       520

Conf:
Pred:
Pred: CCCCCCCCCCCCCCCCCCCCCCCCCCCCCCCCEEEEEEEE
  AA: ADAVSSSTSINDTNRTSLPSPSPDSEHPSAWPIEIEQNAT
                530       540       550       560
```

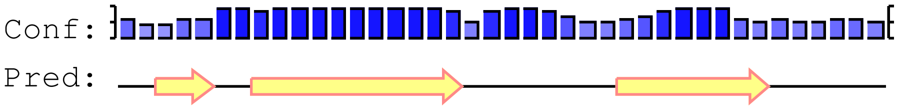

```
Pred: CCEEECCEEEEEEEEEEEECCCCCCCCEEEEEEEEECCCCCC
  AA: ASWTKQGKTYRRYAVTVTNRSPKTVHELHIGISKLYGQVW
                  570       580       590       600
```

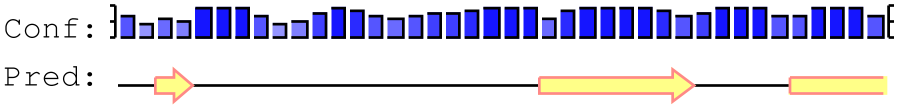

```
Pred: CCEECCCCCCCCCCCCCCCCCCEEEEEEEEECCCCCEEEEE
  AA: GVDKARYGYVLPSWIPSLLAGKSAVFVYVQAAPPADVWVT
                  610       620       630       640
```

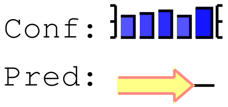

```
Pred: EEEEC
  AA: GYKLI
```

Legend:

= helix   Conf: = confidence of prediction (– to +)

= strand   Pred: predicted secondary structure

= coil   AA: target sequence
